# Unraveling tuberculosis patient cluster transmission chains: integrating WGS-based network with clinical and epidemiological insights

**DOI:** 10.3389/fpubh.2024.1378426

**Published:** 2024-05-20

**Authors:** Darja Sadovska, Iveta Ozere, Ilva Pole, Jānis Ķimsis, Annija Vaivode, Anda Vīksna, Inga Norvaiša, Ineta Bogdanova, Viktorija Ulanova, Valentīna Čapligina, Dace Bandere, Renāte Ranka

**Affiliations:** ^1^Laboratory of Molecular Microbiology, Latvian Biomedical Research and Study Centre, Riga, Latvia; ^2^Centre of Tuberculosis and Lung Diseases, Riga East University Hospital, Upeslejas, Latvia; ^3^Department of Infectology, Riga Stradiņš University, Riga, Latvia; ^4^Department of Pharmaceutical Chemistry, Riga Stradiņš University, Riga, Latvia

**Keywords:** tuberculosis, WGS, transmission, network, recurrence, reactivation, reinfection

## Abstract

**Background:**

Tuberculosis remains a global health threat, and the World Health Organization reports a limited reduction in disease incidence rates, including both new and relapse cases. Therefore, studies targeting tuberculosis transmission chains and recurrent episodes are crucial for developing the most effective control measures. Herein, multiple tuberculosis clusters were retrospectively investigated by integrating patients’ epidemiological and clinical information with median-joining networks recreated based on whole genome sequencing (WGS) data of *Mycobacterium tuberculosis* isolates.

**Methods:**

Epidemiologically linked tuberculosis patient clusters were identified during the source case investigation for pediatric tuberculosis patients. Only *M. tuberculosis* isolate DNA samples with previously determined spoligotypes identical within clusters were subjected to WGS and further median-joining network recreation. Relevant clinical and epidemiological data were obtained from patient medical records.

**Results:**

We investigated 18 clusters comprising 100 active tuberculosis patients 29 of whom were children at the time of diagnosis; nine patients experienced recurrent episodes. *M. tuberculosis* isolates of studied clusters belonged to Lineages 2 (sub-lineage 2.2.1) and 4 (sub-lineages 4.3.3, 4.1.2.1, 4.8, and 4.2.1), while sub-lineage 4.3.3 (LAM) was the most abundant. Isolates of six clusters were drug-resistant. Within clusters, the maximum genetic distance between closely related isolates was only 5–11 single nucleotide variants (SNVs). Recreated median-joining networks, integrated with patients’ diagnoses, specimen collection dates, sputum smear microscopy, and epidemiological investigation results indicated transmission directions within clusters and long periods of latent infection. It also facilitated the identification of potential infection sources for pediatric patients and recurrent active tuberculosis episodes refuting the reactivation possibility despite the small genetic distance of ≤5 SNVs between isolates. However, unidentified active tuberculosis cases within the cluster, the variable mycobacterial mutation rate in dormant and active states, and low *M. tuberculosis* genetic variability inferred precise transmission chain delineation. In some cases, heterozygous SNVs with an allelic frequency of 10–73% proved valuable in identifying direct transmission events.

**Conclusion:**

The complex approach of integrating tuberculosis cluster WGS-data-based median-joining networks with relevant epidemiological and clinical data proved valuable in delineating epidemiologically linked patient transmission chains and deciphering causes of recurrent tuberculosis episodes within clusters.

## 1 Introduction

Tuberculosis (TB) is an infectious disease caused by the *Mycobacterium tuberculosis* (*Mtb*) complex belonging mycobacteria. It is still a devastating public health threat worldwide, and the second leading cause of death from a single infectious agent, after coronavirus disease COVID-19. According to the World Health Organization (WHO), 7.5 million people were newly diagnosed with active TB in 2022, which is the highest single-year number since the onset of the WHO TB monitoring program nearly two decades ago, and it reflected the global recovery in access to healthcare providers which was disrupted during the two-year COVID-19 pandemic (1). The reduction of TB incidence rate, including both new and recurrent cases, is one of the main WHO End TB Strategy objectives; however, the incidence rate reduction of only 8.7% in the period 2015–2022 was documented, while the End TB defined milestone was a 50% reduction by 2025 (1, 2). Therefore, it is crucial to understand TB transmission patterns and dynamics in countries with different-level incidence rates, precisely track the transmission chains, decipher recurrent TB causes, and define host- and pathogen-related contributing factors for transmission and recurrence events to achieve adequate disease management. This knowledge is particularly important for drug-resistant TB case control since it appears that drug resistance is more likely to be transmitted than acquired (3). Thus, in-depth characterization of TB clusters and recurrent TB cases by using epidemiological and clinical data combined with *Mtb* isolate molecular analysis is highly important.

Contact investigation is a systematic non-molecular epidemiological approach to studying TB transmission, which is an important component of TB control programs in low-incidence countries. It is centered on an index patient—the initial new or recurrent TB case—and is aimed at identifying yet undiagnosed active TB cases and individuals harboring latent *Mtb* infection. Notably, an index patient is not necessarily a source case, who transmitted TB infection to one or more individuals (4–6). An opposite process—reverse contact investigation, also known as source case investigation, —is often conducted mostly to identify a source case for a TB-infected child (6, 7). However, established epidemiological links need to be complemented by molecular *Mtb* typing results demonstrating that *Mtb* isolates obtained from these patients share the same genotyping pattern. If both conditions are met, TB cases are considered to be involved in the same TB transmission chain also commonly referred to as “TB cluster” (8).

Conventional genotyping methods, such as mycobacterial interspersed repetitive unit-variable number of tandem repeat (MIRU-VNTR), insertion sequence *6110* restriction fragment length polymorphism (RFLP), and spoligotyping, have been successfully applied for this purpose for decades (9–12). Unfortunately, these methods lack sufficient discriminative power to confirm recent person-to-person transmission or distinguish remotely related *Mtb* strains belonging to the same genotype, which can lead to false clustering (13). These genotyping methods were also used to determine the causes of recurrent TB episodes (14–17). TB recurrence is caused by either endogenous reactivation of previously acquired *Mtb* infection, or exogenous reinfection with a new *Mtb* strain (18). As in TB cluster investigation, the application of any conventional genotyping method for recurrent TB case analysis also has limitations and is only helpful in distinguishing reinfection with *Mtb* strain of distinct genotype.

Whole genome sequencing (WGS) of *Mtb* has demonstrated a higher resolution when investigating TB transmission chains (13, 19–22) and recurrent TB episodes (23–25) than conventional genotyping methods. The application of WGS allows the detection of differing single nucleotide variants (SNVs) and therefore - the measurement of genetic relatedness between studied *Mtb* isolates. Moreover, the pattern of SNV accumulation is thought to indicate the direction of TB transmission within clusters (19, 26). Currently, the thresholds of pairwise SNV distances between isolates are widely used for identifying the epidemiologically relevant TB clusters (≤12 SNVs), inferring recent transmission events (≤5 SNVs) (19–21, 26–28), and distinguishing endogenous reactivation (≤5 up to 12 SNVs) from exogenous reinfection (≥100 SNVs) (23–25, 29, 30). Although thresholds suggested for the cause determination of recurrent TB cases mostly corresponded with the results of different conventional genotyping methods, inconclusive recurrent TB cases when SNV distances between *Mtb* isolates laid between the defined thresholds (<100 SNVs) were also reported and classified as reinfections (25, 31). Furthermore, results of recently conducted studies also demonstrated, that analyzing pairwise SNV distances to determine the cause of recurrent TB episodes is insufficient to recognize reinfection with closely related *Mtb* strain. This observation is particularly important in the case of recurrent TB patients who have been involved in local TB transmission chains with epidemiologically closely linked individuals (32–34).

Latvia, a Baltic state in Northern Europe, is a low-moderate TB incidence country, however, it is one of the WHO priority countries in the European region due to relatively high drug-resistant TB prevalence (35). In 2022, a total number of 350 new and recurrent TB cases was reported (19 per 100,000 population) and 39 (11.1%) of them were multidrug- (MDR) or rifampicin-resistant (36). The widespread MDR and pre-extensively drug-resistant (pre-XDR) *Mtb* strains have been a significant concern for Latvian healthcare authorities for decades now, and numerous *Mtb* genotypes mostly belonging to the Lineages 2 and 4 were identified among both drug-resistant and susceptible isolates acquired from TB patients in various studies, emphasizing the TB as a major health threat for Latvian population (37–40).

In the present study, we recreated median-joining networks of multiple TB clusters based on the differing SNVs between *Mtb* isolates and interpreted them using previously conducted epidemiological investigation results and relevant clinical data to (1) verify if the known index case was also the initial source case; (2) identify the source cases for pediatric TB patients involved in studied clusters and compare acquired results with epidemiological data; (3) determine the causes of recurrent TB cases within clusters; (4) identify contributing and interfering factors for transmission chain delineation in the studied sample set. In addition, we also analyzed the genetic diversity of *Mtb* strains within clusters and between all *Mtb* isolates belonging to the same genotype and described the SNV accumulation pattern by comparing SNV profiles of closely related *Mtb* strains.

## 2 Materials and methods

### 2.1 Study population

In this study, we retrospectively investigated previously defined epidemiologically linked and *Mtb*-isolate-genotype-matched TB patient clusters that were first discovered during the source case investigation conducted for pediatric TB patients, who were diagnosed in 2003–2016 and had culture-confirmed TB disease. Herein, we used the terms “child” or “pediatric” in reference to those studied patients who were diagnosed with active TB when they were under 18 years old, while pediatric patients from 13 years old were also referred to as “adolescents” (41). Surveillance of these TB clusters, contact tracing, and conventional *Mtb* isolate genotyping of all active TB cases potentially involved in these transmission chains were conducted until the end of 2019. All clusters consisted of both pediatric and adult active TB patients. Patients involved in this study were admitted to the Riga East University Hospital’s Centre of Tuberculosis and Lung Diseases, the nationwide TB diagnostics and treatment center, Daugavpils Regional Hospital, Liepaja Regional Hospital, or Rezekne Hospital depending on the patient’s place of residence and were diagnosed with active TB disease in the period 2002–2019.

Active TB cases epidemiologically linked to pediatric TB patients were classified according to the degree of exposure as recommended previously (4): (1) individuals who shared the same household with a potential source of infection were classified as close household contacts; (2) individuals, who had regular, prolonged contact with a potential source of infection but did not live in the same household, for instance, relatives, neighbors, close friends, and classmates, were classified as close nonhousehold contacts; (3) individuals, who spent less time with a potential source of infection, for instance, school staff, passengers regularly using the same public transport, and acquaintances, were classified as casual contacts. Patient medical records were studied retrospectively to define the geographical distribution of each TB cluster and to collect clinical data on studied TB patients’ biological sex, diagnosis, age at the time of diagnosis, sputum smear microscopy results, specimen collection date for mycobacterial isolation, phenotypic drug susceptibility testing (pDST) results, and epidemiological links between patients within clusters.

We confirm that all experimental protocols were approved by the Riga Stradiņš University Research Ethics Committee (Nr. 6-1/06/12; 28.05.2020), the Centre for Disease Prevention and Control of Latvia (Nr.14; 22.07.2020), and the Science Department of Riga East University Hospital (Nr. ZD/08-06/01–20/215; 10.09.2020). Written informed consent was waived by the Riga Stradiņš University Research Ethics Committee due to the retrospective nature of this study as only previously acquired mycobacterial DNA samples and patients’ medical records were investigated in this study. We can confirm that all methods were carried out following relevant guidelines and regulations in the Declaration of Helsinki.

### 2.2 Geospatial analysis

Data regarding the cities, parishes, and municipalities of clustered patient residence addresses and documented locations where physical contact occurred between these individuals, for instance, educational institutions, were obtained from patients’ medical records. The geospatial distribution of each cluster was visualized in Tableau Desktop software (v2023.3.0). Standard calibration was applied for five points (Riga (C), Liepaja (D), Tukums (L), Salaspils (P), and Ventspils (I)), and custom calibration was used for other locations.

### 2.3 Phenotypic drug susceptibility testing, mycobacterial DNA extraction, and spoligotyping

*Mtb* cultivation, pDST, mycobacterial DNA extraction, and *Mtb* isolate spoligotyping for all investigated TB cases were conducted at the clinical laboratory of the Centre of Tuberculosis and Lung Diseases, while sputum smear microscopy was performed at the hospital, where each patient was admitted. *Mtb* cultivation and pDST for each patient were performed at the time of diagnosis, while *Mtb* DNA extraction and spoligotyping were carried out during source case investigation for pediatric TB patients and continued during the following contact tracing for each active TB patient potentially involved in one of these clusters.

Mycobacterial DNA samples were extracted from *Mtb* cultures grown on Lowenstein-Jensen (LJ) media according to the cetyltrimethylammonium bromide protocol (42). If the source case investigation indicated that past TB patients could be involved in one of the studied transmission chains, their *Mtb* isolates were regenerated from the laboratory’s culture bank. Afterward, spoligotyping was conducted using commercially available reagent kits (Isogen Life Science, Netherlands; later—Ocimum Biosolutions, India) based on the previously published protocol (43). Spoligotype SIT numbers were inferred using the SITVIT2 database (available at http://www.pasteur-guadeloupe.fr:8081/SITVIT2/). Only epidemiologically linked TB patients with identical *Mtb* isolate spoligotype patterns were subjected to WGS and detailed transmission chain analysis. Analyzed TB cases in each cluster were arranged according to the specimen collection date in chronological order starting with the known index case.

Phenotypic drug resistance profiles of clustered patients’ *Mtb* isolates were compiled based on available pDST reports in hospital medical records. The pDST was conducted using either Bactec Mycobacterial Growth Indicator Tube (MGIT) 960 system (Becton Dickinson, Heidelberg, Germany) or LJ media according to up-to-date WHO guidelines, which were in effect at the time of test performance. Ofloxacin, moxifloxacin, and levofloxacin were analyzed as a single “fluoroquinolone” group.

Among isolates analyzed in this study, the critical concentrations for Bactec MGIT 960 system (liquid media) were 1 μg/mL (years 2008–2019) and 2 μg/mL (years 2002–2004) for rifampicin, 0.1 μg/mL for isoniazid, 5 μg/mL (years 2008–2019) and 7.5 μg/mL (years 2002–2004) for ethambutol, 100 μg/mL for pyrazinamide, 2 μg/mL (years 2009–2017) for ofloxacin, 0.25 μg/mL (years 2009–2019), 0.5 μg/mL (year 2015), 1 μg/mL (year 2019), and 2 μg/mL (year 2015) for moxifloxacin, 1 μg/mL (year 2019), and 2 μg/mL (year 2015) for levofloxacin, 1 μg/mL (years 2008–2016) and 6 μg/mL (years 2002–2004) for streptomycin, 1 μg/mL for amikacin (years 2009–2019), 2.5 μg/mL (years 2009–2019) for capreomycin, 2.5 μg/mL (years 2015–2019) for kanamycin, 1 μg/mL (year 2019) for bedaquiline, 1 μg/mL (year 2019) for clofazimine, 1 μg/mL (years 2015–2019) for linezolid, and 0.06 μg/mL (year 2019) for delamanid. The critical concentrations for LJ media were 40 μg/mL (years 2003–2017) for rifampicin, 0.2 μg/mL (years 2004–2017) and 1 μg/mL (years 2003–2017) for isoniazid, 2 μg/mL (years 2003–2017) for ethambutol, 4 μg/mL (years 2009–2017) for ofloxacin, 4 μg/mL (years 2003–2013) for streptomycin, 30 μg/mL (years 2009–2019) for amikacin, 40 μg/mL (years 2009–2019) for capreomycin, 30 μg/mL (years 2003–2017) for kanamycin, 40 μg/mL (years 2009–2017) for ethionamide, 1 μg/mL (years 2009–2019) for para-aminosalicylic acid, and 30 μg/mL (years 2009–2019) for D-cycloserine.

For those *Mtb* isolates, when pDST was conducted by both methods, results acquired from testing on both solid and liquid media were compared and combined in the phenotypic drug resistance profile. If results were mismatched, and the *Mtb* isolate demonstrated resistance to any medication in only one of the performed tests, the isolate was considered resistant to this medication.

### 2.4 Whole genome sequencing, and bioinformatic data processing

Mycobacterial DNA samples were quantified using the Qubit dsDNA Broad Range Assay Kit and Qubit 3.0 Fluorometer (Invitrogen, Thermo Fisher Scientific, United States). 1 μg DNA input was used for the preparation of sequencing libraries. DNA samples were physically sheared down into 200 base pairs fragments with an S220 Focused-ultrasonicator (Covaris, United States), and single-end fragment libraries were prepared using the Ion Plus Fragment Library Kit (Ion Torrent, Thermo Fisher Scientific, United States) according to the manufacturer’s protocol. Before sequencing, DNA fragment libraries were quantified using the Qubit dsDNA High Sensitivity Assay Kit, while fragment length distribution was measured with the High Sensitivity DNA Kit and 2100 Bioanalyzer (Agilent Technologies, United States). DNA libraries were sequenced on an Ion Proton System (Ion Torrent, Thermo Fisher Scientific, United States). Reads of a maximum of 200 base pairs were produced.

The bioinformatics pipeline applied for WGS data of studied *Mtb* isolates was described previously (40). Sequencing data analysis, including quality control and filtering, mapping to a reference sequence, and variant site calling was performed on the Galaxy web platform at https://usegalaxy.org public server (44). The sequence of the inferred *Mtb* complex’s most recent common ancestor combined with the annotation of the H37Rv sequence (*Mtb* ancestral genome; GenBank NC000962.3) shared previously was used as a reference sequence for WGS read mapping and phylogenetic analyses (45). We applied a threshold of 12 SNVs between *Mtb* isolates to confirm the involvement of each TB case in one of the studied clusters. To determine differing SNVs between clustered *Mtb* isolates, variant sites containing VCF files were merged using VCFcombine (v1.0.0), and all differences were checked manually using Integrative Genomics Viewer (IGV; v2.16.2). Heterozygous bases detected among differing SNVs between *Mtb* isolates within clusters were analyzed in this study, and the variant was considered homozygous if its allele frequency (AF) was ≥90%.

Sequence alignment files (BAM) generated by the snippy tool (v4.6.0) were used as TB-Profiler software inputs (v4.4.1) to determine *Mtb* sub-lineages and detect genetic variants in genes associated with *Mtb* drug resistance (46). Variants with AF ≥ 10% and a minimum coverage depth of four sequencing reads per base were called. The significance of all detected variants was assessed using the catalog of *Mtb* variants and their association with drug resistance developed by WHO (47). Variants of yet uncertain significance (grading group 3) were interpreted as resistance-associated if they had been previously detected in drug-resistant strains.

### 2.5 Phylogenetic analyses and delineation of transmission chains

Multiple sequence core alignments which included all identified SNV sites were created using the snippy-core tool (v4.6.0). The outgroup-rooted maximum likelihood phylogeny based on the core alignment of the whole dataset was estimated using IQ-TREE 2 (v2.0.7) software with 1,000 ultrafast bootstrap replications and applying the ModelFinder algorithm for best-fit evolutionary model selection (48–50). For this analysis, heterozygous bases were encoded as “N” in the alignment, while in the case of identified two-strain coinfection (K3), sequences of both strains were recreated based on *Mtb* isolate SNV profiles of two other TB patients involved in this cluster and presented in generated phylogeny. The phylogenetic tree was annotated on the iTOL public server (v6.9; available at https://itol.embl.de/) displaying bootstrap support of more than 83.5% on the branches.

Median-joining networks based on the core alignments of all *Mtb* isolates belonging to the same sub-lineage were constructed in PopART software (v1.7) (51). The *Mtb* ancestral genome served as a reference point for the examination of genetic change direction in the networks. Each network node represented TB episodes of one or more individuals infected with *Mtb* strains separated by no SNVs further referred to as “identical.” Network nodes were scaled according to the number of identical *Mtb* isolates, while numbers in brackets along network branches indicated the number of differing SNVs between nodes. The genetic distance between *Mtb* isolates was calculated following the shortest path in the median-joining network.

In this study, we also attempted to determine the direction of TB infection transmission between clustered patients by simultaneously analyzing SNV distances between acquired *Mtb* isolates, patients’ diagnoses, sputum smear microscopy results, time intervals between specimen collection dates within clusters considering the infection incubation period of at least 14 days and established epidemiological links between patients. For this purpose, we also operated with network distances—the number of nodes traversed on the shortest path between two TB cases. Patients having extrapulmonary TB forms were considered non-contagious, while patients with positive sputum smear microscopy were considered more likely to transmit TB infection (52).

### 2.6 Data statistical analysis

All calculations and statistical analyses were performed in RStudio software (2023.12.1). A level of significance α = 0.05 was chosen for all performed tests. The average patient number per cluster and the average time between specimen collection from the first and last TB patients were reported for clusters belonging to the same sub-lineage. The distribution normality of patient age at the time of diagnosis, sequencing data quality score, and coverage depth were checked using three approaches: construction of both quantile diagram and boxplot and performing Shapiro–Wilk test; the median and interquartile range (IQR) were calculated for these data. The chi-square goodness of fit test with Bayes factor upper bound and effect size calculation was performed to assess the occurrence distribution of recently acquired SNV effects.

## 3 Results

### 3.1 Characteristics of patients with tuberculosis in this study

In total, 18 clusters (A-R) involving 115 TB patients were studied (Table 1). The number of patients per cluster varied from three to 17, and 10 patients experienced recurrent active TB episodes. From one patient *Mtb* isolate could not be obtained, 13 *Mtb* isolates were excluded from the study based on the spoligotyping results, and two more *Mtb* isolates were not available at the time of WGS performance. Thereby, 109 *Mtb* isolates acquired from 100 TB patients nine of whom had recurrent TB episodes were subjected to WGS. Each isolate represented one active TB case.

**Table 1:** Characteristics of the studied TB clusters.

Cluster	Lineage	Sub-lineage	SIT (spoligotype family)	Drug resistance type	No. of epidemiologically linked TB patients (*n* = 115)	No. of studied *Mtb* isolates with identical spoligotypes (*n* = 109)	Years
A	4	4.3.3 (LAM)	42 (LAM9)	S	17*	14	2005–2016
B				Pre-XDR	4	3	2009
C			254 (LAM-RUS)	S	5*	6	2010–2019
D				S	9**	6	2004–2015
E				S	12*	13	2008–2017
F				S	5	5	2016–2017
G		4.1.2.1 (Haarlem)	283 (H1)	Hr	4*	5	2008–2015
H			50 (H3)	Other	8**	10	2006–2016
I				S	7	6	2010–2016
J		4.8 (mainly T)	53 (T1)	S	4	3	2008–2015
K				Hr/MDR	4	4	2011–2013
L				S	3*	4	2014–2019
M			280 (T1-RUS2)	S	3	3	2015
N		4.2.1 (Ural)	3340 (Ural-1)	S	11	8	2002–2010
O				S	3	3	2012–2013
P	2	2.2.1 (Beijing)	1 (Beijing)	S	6*	7	2011–2014
Q				Hr	6	6	2014–2016
R				Hr	4	3	2013–2017

*One patient had a recurrent TB episode. **Two patients had a recurrent TB episode. S, sensitive; Hr, isoniazid-resistant; MDR, multidrug-resistant; Pre-XDR, pre-extensively drug-resistant.

Eighty of 109 sequenced *Mtb* isolates (73.4%) were obtained from 73 adult TB patients, and 29 isolates (26.6%)—from 29 pediatric TB patients. Sixty-four adults and 27 children experienced a single TB episode: there were 40 males (62.5%) and 24 females (37.5%) among adult patients, and 11 males (40.7%) and 16 females (59.3%) among pediatric patients. In nine cases of TB recurrence, three male and four female patients were adults during both TB episodes, and two more male patients had their initial TB episode in adolescence. The median age at the time of diagnosis calculated per each TB episode was 33.5 years (IQR 26.75–43) for adult patients and 14 years (IQR 10–16) for pediatric patients. Pulmonary TB was the most common disease form in both adult and pediatric populations, including both episodes of all recurrent TB cases. In the adult population, there were 77 (77/80, 96.25%) pulmonary TB episodes, two (2/80, 2.5%) tuberculous pleurisy episodes, and one episode (1/80, 1.25%) of simultaneous pulmonary TB and peripheral tuberculous lymphadenitis. In the pediatric population, there were 25 (25/29, 86.2%) pulmonary TB episodes, two (2/29, 6.9%) tuberculous pleurisy episodes, one (1/29, 3.4%) tuberculous meningitis episode, and one episode of simultaneous pulmonary TB and tuberculous pleurisy. Detailed information is included in Supplementary material S1.

**Figure 1:**
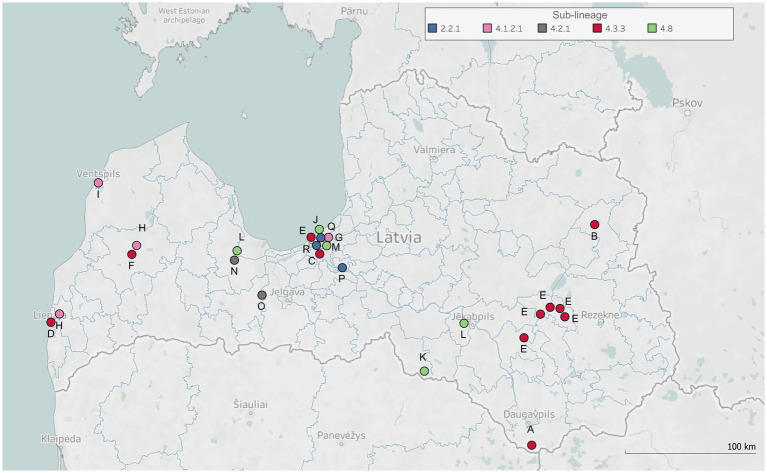
Geospatial distribution of studied tuberculosis clusters. The map displays geographical locations (i.e., cities, municipalities, or parishes in Latvia) representing the residence addresses of clustered patients, or other documented locations where physical contact occurred between these individuals and TB infection might be transmitted. Each dot on the map corresponds to one of these locations. The dots are annotated with symbols corresponding to each studied TB cluster (labeled A-R) and colored according to the determined sub-lineages of clusters’ *Mtb* isolates.

In most cases, patients involved in the same cluster resided in the same city (clusters B, C, D, F, G, I, J, M, N, P, Q, and R) or parish (clusters A, K, and O), and in case of three clusters (E, H, and L) a larger geographical area was covered (Figure 1). Patients involved in six clusters (C, G, J, M, Q, and R) and some patients of cluster E lived in the capital of Latvia, Riga, which is a residential city for a third of the Latvian population (data from Central Statistical Bureau of Latvia, year 2023). Investigated clusters mostly included only the child’s family members living either in the same (K and O) or different households (B, D, F, G, L, M, P, and R), while other clusters involved both child’s family and family friends (C and N), classmates (E, H, and I), school staff (E), neighbors (N and Q), and acquaintances (A and J). Therefore, close household and close nonhousehold contact types were the most prevalent among investigated clusters, and both contact types were determined between TB patients of 10 clusters (D, F, G, H, I, L, N, P, Q, and R). Patients of three clusters (C, K, and O) had close household contact, and patients of two clusters (B and M) had close nonhousehold contact. In two clusters (A and E), close household, close nonhousehold, and casual contact types were determined, and in one cluster (J), both close household and casual contact types were identified.

### 3.2 Genotyping, drug susceptibility testing, and maximum-likelihood phylogeny of the studied *Mycobacterium tuberculosis* isolates

For all sequenced *Mtb* DNA samples, the median base quality score was 25.9 (IQR 25.8–26.1), and the median reference genome coverage depth was 65.1 reads per base (IQR 43.3–77.3). All sequenced isolates were kept for further analysis.

WGS-based genotyping and spoligotyping results were concordant. However, spoligotyping demonstrated greater discriminatory power in this dataset as in most cases multiple spoligotypes representing the same TB-Profiler-assigned sub-lineage were identified among studied isolates. *Mtb* isolates of investigated clusters belonged to the Lineages 2 and 4 (Table 1). Sub-lineage 4.3.3, comprising SIT42 and SIT254 spoligotypes, was determined for *Mtb* isolates in six clusters (A-F). Other Lineage 4 sub-lineages were 4.1.2.1 (SIT283 and SIT50), 4.8 (SIT53 and SIT280), and 4.2.1 (SIT3340) which were identified for *Mtb* isolates of three (G-I), four (J-M), and two (N and O) clusters, respectively. Lineage 2 was represented by sub-lineage 2.2.1 (SIT1) and it was determined for three clusters (P-R).

**Figure 2:**
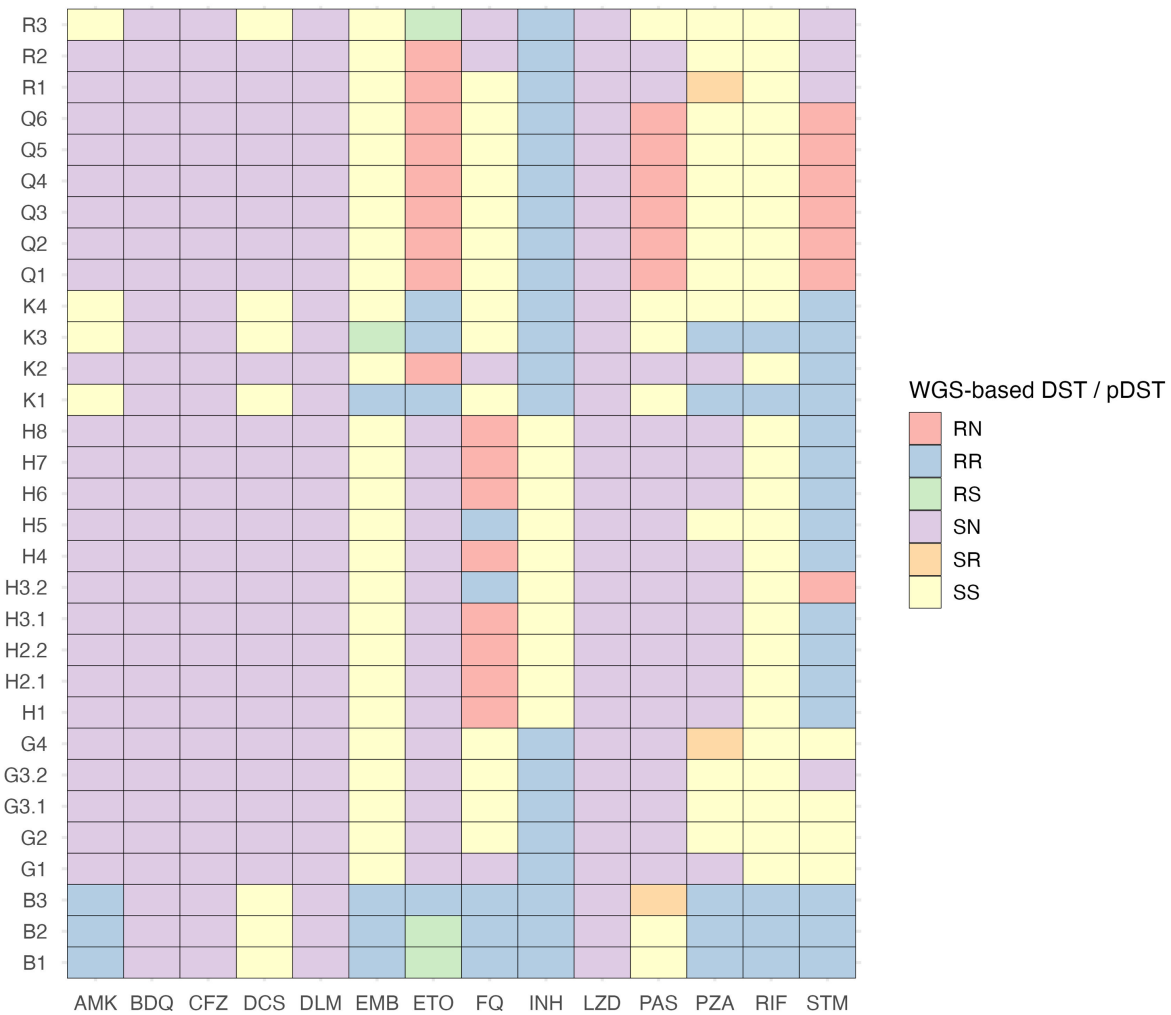
Comparison of phenotypic and WGS-based drug susceptibility testing results of drug-resistant *M. tuberculosis* isolates. AMK, amikacin; BDQ, bedaquiline; CFZ, clofazimine; DCS, D-cycloserine; DLM, delamanid; DST, drug susceptibility testing; EMB, ethambutol; ETO, ethionamide; FQ, fluoroquinolones; INH, isoniazid; LZD, linezolid; N, drug susceptibility testing results are not available; PAS, para-aminosalicylic acid; pDST, phenotypic drug susceptibility testing; PZA, pyrazinamide; R, resistant; RIF, rifampicin; S, sensitive; STM, streptomycin.

We compared phenotypic (Supplementary material S2) and WGS-based DST (Supplementary material S3) results to present the most accurate drug resistance profiles of studied *Mtb* isolates. According to WGS, no resistance-associated variants were detected among *Mtb* isolates of 12 clusters (A, C, D, E, F, I, J, L, M, N, O, and P), and pDST data confirmed the drug susceptibility of these isolates. WGS data analysis results indicated the presence of resistance-associated variants in *Mtb* isolates of clusters B, G, H, K, Q, and R. WGS-predicted drug resistance profiles among isolates belonging to the same cluster were identical, except for isolates of cluster K, that demonstrated two different drug-resistance profiles. In drug-resistant *Mtb* isolates, resistance-associated variants for rifampicin, isoniazid, ethambutol, pyrazinamide, fluoroquinolones, streptomycin, amikacin, capreomycin, kanamycin, ethionamide, and para-aminosalicylic acid were detected, and there were seven disagreements between phenotypic and WGS-based DST results (Figure 2). According to the WHO classification of drug-resistant TB (53, 54), *Mtb* isolates of clusters G, Q, and R were isoniazid-resistant (Hr), cluster B was pre-XDR, and the drug resistance type of cluster H was defined as “other” since *Mtb* isolates were only fluoroquinolone- and streptomycin-resistant. In cluster K, both Hr and MDR *Mtb* isolates were present.

**Figure 3:**
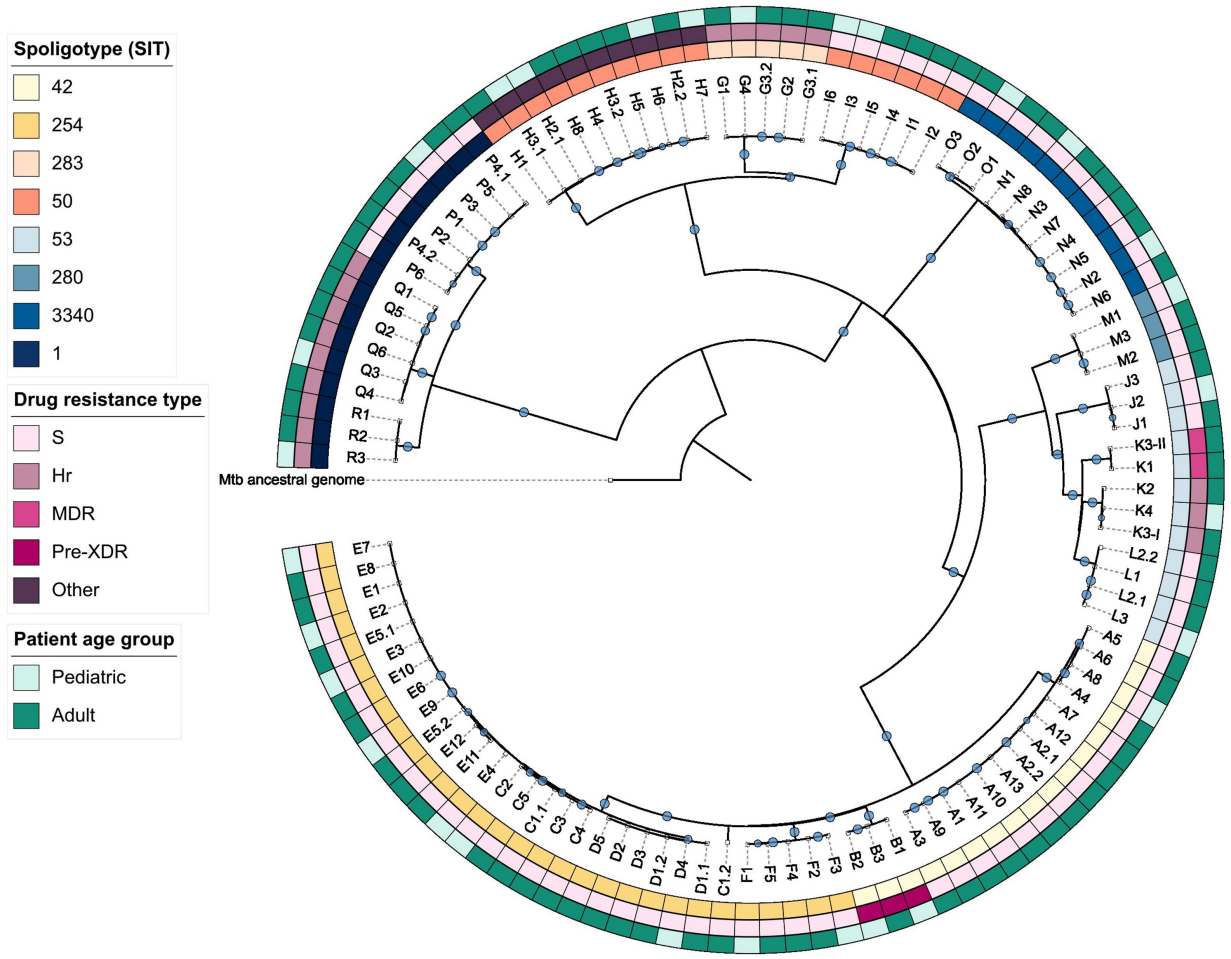
Maximum-likelihood phylogeny of the studied dataset. Hr, isoniazid-resistant; MDR, multidrug-resistant; Pre-XDR, pre-extensively drug-resistant; S, sensitive.

The created maximum-likelihood phylogeny of the dataset (Figure 3) indicated that within the Beijing (SIT1), Ural (SIT3340), and LAM (SIT254 and SIT42) families *Mtb* isolates exhibit greater genetic relatedness than in other clades. Clusters E, D, and all but one isolates of cluster C (case C1.2) formed a monophyletic group; however, no common ancestors between any clusters could be identified.

### 3.3 Transmission chain analysis and recurrent tuberculosis cause determination

#### 3.3.1 Sub-lineage 4.3.3

**Figure 4:**
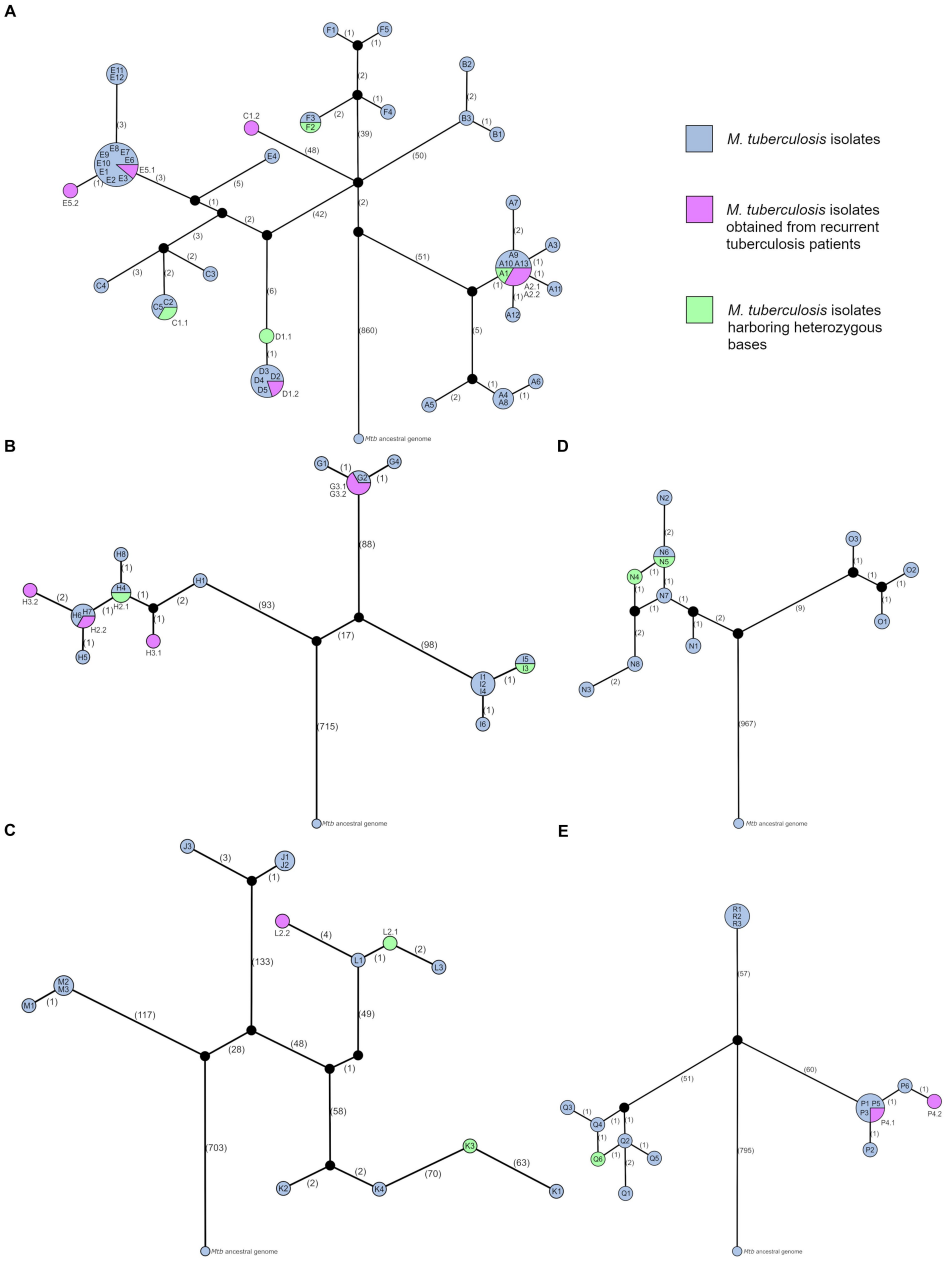
Median-joining networks of studied tuberculosis clusters. Networks illustrate genetic distances between *Mtb* isolates obtained from clustered patients with active TB and belonging to sub-lineages **(A)** 4.3.3 (LAM; clusters A–F), **(B)** 4.1.2.1 (Haarlem; clusters G–I), **(C)** 4.8 (T; clusters J–M), **(D)** 4.2.1 (Ural; clusters N and O), and **(E)** 2.2.1 (Beijing; clusters P–R). Using the *Mtb* ancestral genome as a reference point, networks demonstrate the direction of genetic changes between isolates. Each network node represents TB episodes of one or more patients infected with identical *Mtb* strains (zero SNV distance). Symbol combinations (i.e., A1) within nodes, and for recurrent TB cases—next to nodes (i.e., A2.1 and A2.2), represent each active TB episode. Network nodes are scaled according to the number of identical *Mtb* isolates, while numbers in brackets along network branches indicate the number of differing SNVs between nodes.

Almost half of the studied *Mtb* isolates belonged to the sub-lineage 4.3.3, and it was identified for 47 isolates in six clusters (A-F). Seventeen *Mtb* isolates obtained from 14 pulmonary TB and two tuberculous pleurisy patients (cases A6 and A8) had SIT42 corresponding pattern (clusters A and B), and 30 *Mtb* isolates obtained from 24 pulmonary TB patients, one tuberculous meningitis patient (case C2), one simultaneous pulmonary TB and peripheral tuberculous lymphadenitis patient (case C5), and one simultaneous pulmonary TB and tuberculous pleurisy patient (case E7) belonged to SIT254 spoligotype (clusters C-F). *Mtb* isolates of all but one clusters were drug-susceptible, while isolates of cluster B were pre-XDR. The average cluster size was 7.2 patients (range 3–13), and the average time between specimen collection from the first and last cluster TB patients was 83.48 months (range 0.07–129.37). The genetic distance range between *Mtb* isolates belonging to two different SIT42 clusters was 105–112 SNVs, between four different SIT254 clusters it was equal to 9–99 SNVs, and between SIT42 and SIT254 *Mtb* isolates the distance range was 90–111 SNVs (Figure 4A). Within the clusters, SNV distances between *Mtb* isolates were 0–10 (A), 1–3 (B), 0–98 (C), 0–1 (D), 0–11 (E), and 0–5 (F).

The network topology of cluster A revealed the possibility of unidentified TB cases belonging to this cluster, including the initial source of infection, as the cluster network split creating two branches with 7–10 SNV distance between *Mtb* isolate sub-groups. The star-like node pattern visualizing the first *Mtb* isolate sub-group suggested the presence of a super-spreader in the common node consisting of six identical *Mtb* isolates, however, no available epidemiological data could support it. Notably, the specimen of index case A1 was obtained 52.43 months earlier than the case A2.1 specimen, which suggests a long latent TB infection period in at least one individual. Furthermore, a heterozygous base g.3471129C > G was called in A1 *Mtb* isolate with AF = 73%, and this variant was harbored by other isolates from this sub-group with AF = 100%, suggesting that A1 was the initial source of infection in this sub-group. One patient in this cluster experienced a recurrent TB episode (cases A2.1 and A2.2). *Mtb* isolates acquired from this patient were identical to isolates of four more patients, one of whom (case A9) was diagnosed with active TB approximately 9 months earlier than case A2.2. Thus, reinfection is thought to be the cause of recurrence. Nine of the 11 TB patients in the first sub-group were only epidemiologically linked by occasionally using the same public transport, and two patients (cases A3 and A7) were family members. However, the network did not indicate the direct transmission event between cases A3 and A7.

The second sub-group of cluster A consisted of three family members (cases A4, A5, and A8), and a person who regularly used the same public transport (case A6). According to the median-joining network, case A4 was the source of infection for pediatric TB case A8, and not case A5 as was suggested during the epidemiological investigation. Notably, case A4 unlike case A5 had negative sputum smear microscopy, however, it was also a potential source of infection for case A6 as demonstrated by the network. Similarly, the network topology of cluster B confirmed that the sputum-smear-negative case B3 was the source of infection for both pediatric TB cases B1 and B2. Furthermore, all patients’ specimens were acquired within 2 days, but secondary cases B1 and B2 differed by 1–2 SNVs from the source case B3.

In cluster C, the network topology indicated that the source of infection causing at least three secondary cases C1.1, C3, and C4 remained unidentified. Moreover, based on patients’ specimen acquisition dates we suspect long latent TB infection periods of at least 20.37 and 64.5 months in cases C4 and C5, respectively. Notably, two heterozygous bases g.887618C > T and g.4169716C > T were detected in the index case C1.1 isolate with AF = 63% and AF = 56%, respectively. These variants were also found in C2 and C5 isolates with AF = 96.3–100%. This finding indicated that case C1.1 was the source of infection for case C5 and pediatric TB case C2. A large genetic distance (98 SNVs) reflected one patient’s reinfection event with a remotely related *Mtb* strain belonging to the same genotype (cases C1.1 and C1.2). It indicated that the source case of the second episode C1.2 was not included in this study. Therefore, case C1.2 can be excluded from the analysis since it is not involved in the studied transmission chain, and cluster isolates exhibit a small genetic distance range of 0–5 SNVs.

According to the cluster D median-joining network, the index case D1.1 was also the initial source case, whose specimen was obtained 123.27 months earlier than the case D2 specimen, therefore we suspect a long latent TB infection in at least this individual. The case D1.1 was separated from others by 1 SNV, however, it harbored two heterozygous bases g.2214615A > G and g.2360875C > A with AF = 46% and AF = 67%, respectively, while in other *Mtb* isolates these variants were detected with AF = 99–100%. Considering very limited *Mtb* isolate genetic variability and close epidemiological link between all involved individuals, the source of infection for the pediatric TB case D4 could not be identified. In this cluster, the index case experienced TB recurrence (cases D1.1 and D1.2). As the *Mtb* isolate of the second episode was identical with four other patient isolates, one of whom was diagnosed approximately 5 months earlier than the case D1.2, reinfection is thought to be a cause of TB recurrence.

The network of cluster E split creating two branches and separating case E4 from others by 8–11 SNV-distance, suggesting that the initial source case was unidentified. Although this cluster involved individuals who came from different geographical areas (Figure 1) and TB infection was transmitted in multiple households, educational institution, and among casual contacts, *Mtb* isolates acquired very limited genetic variability. *Mtb* isolates of nine TB cases were identical, including the index case E1, whose specimen was acquired 56.37 months earlier than E2, E3, and E4 specimens suggesting long periods of latent TB infection in multiple individuals. According to epidemiological data, case E1 was the potential source of infection for E2, E3, E4, and E5.1, and E3 transmitted TB infection further within the educational institution (cases E6, E7, E8, and E9). Unfortunately, a more detailed transmission chain could not be obtained based on WGS data due to tight specimen acquisition timeline between some patients and possible unidentified active TB cases belonging to this cluster, thus sources of infection for five pediatric TB patients (cases E2, E3, E4, E7, and E9) could not be precisely identified. One patient in this cluster experienced a recurrent TB episode (cases E5.1 and E5.2). Although recurrent TB isolates exhibited the difference of only 1 SNV, reinfection is thought to be the cause of recurrence, as the first episode had identical *Mtb* genomes with eight other cases, and, based on the specimen collection dates and epidemiological data, at least one case (E10) could be the source of infection for the second episode.

Notably, a small genetic distance range of 9–16 SNVs was identified between *Mtb* isolates of clusters D, and E, and all but one isolates of cluster C. The periods of TB infection transmission within these clusters overlapped (Table 1), and although one of the documented cluster E geographical locations (Riga) matched the residence place of patients involved in cluster C, location of the cluster D was approximately 200 kilometers away. Nevertheless, considering the small genetic distances between *Mtb* isolates and hypothetical unidentified initial source cases, we suggest that closely related *Mtb* strains were transmitted within a wide geographical area in Latvia.

The network topology of cluster F revealed that multiple TB cases belonging to this cluster remained unidentified, as only two cases (F2 and F3) were directly connected, and a path between all other TB cases included one negative network step overturning the possibility of direct transmission events. This cluster involved two pediatric TB patients (cases F1 and F3). Cases F2 and F3 had identical *Mtb* genomes, however, two heterozygous bases were called in F2 isolate: g.1868543C > T and g.4000852G > A were found with AF = 47% and AF = 48%, respectively, while in F3 these variants were detected with AF = 100%. This finding indicated the TB transmission from case F2 to F3. It supported the epidemiological data suggesting that F2 was the potential source of infection for the pediatric TB case F3. In contrast, the source of infection for another pediatric TB case F1 could not be identified.

#### 3.3.2 Sub-lineage 4.1.2.1

Among 21 *Mtb* isolates belonging to the sub-lineage 4.1.2.1, 16 isolates acquired from 13 pulmonary TB patients and one tuberculous pleurisy patient (case H5) had SIT50 spoligotype corresponding pattern (clusters H and I), while SIT283 spoligotype was determined for five isolates obtained from four pulmonary TB patients (cluster G). *Mtb* isolates of cluster G were Hr, cluster H was fluoroquinolone- and streptomycin-resistant, and cluster I was drug-susceptible. The average cluster size was 6 patients (range 4–8), and the average time between specimen collection from the first and last cluster TB patients was 94.65 months (range 75.63–121.13). The genetic distance range between *Mtb* isolates belonging to two different SIT50 clusters was 208–215 SNVs, and between SIT50 and SIT283 *Mtb* isolates the distance range was 186–205 SNVs (Figure 4B). *Mtb* isolates within clusters were separated by 0–1 SNVs (G and I) and 0–6 SNVs (H).

The median-joining network of cluster G revealed, that *Mtb* isolate of the index case G1 harbored 1 differing SNV compared to the initial network node, which included TB cases G2, G3.1, and G3.2, whose specimens were acquired at least 16.07 months (case G2) later. Therefore, a latent infection period in at least one of these individuals, and possible unidentified active TB cases that could be potential sources of infection for all individuals involved is suspected. According to the specimen acquisition timeline, cases G2 and G3.1 are potential sources of infection for pediatric TB case G4. Considering the positive sputum smear microscopy of case G3.1 and the tighter epidemiological link due to sharing the same household with case G4, we suggest that G3.1 was the source of infection, supporting the epidemiological investigation results. One patient in this cluster experienced recurrent TB episode (cases G3.1 and G3.2). *Mtb* isolates obtained from this patient were identical, and no potential source of infection for the second episode could be identified based on the network topology, epidemiological data, and specimen acquisition timeline, therefore reactivation as a recurrent TB cause is plausible.

The network topology of cluster H corresponded to the specimen acquisition timeline, demonstrating the initial TB infection transmission within a household, and further spreading within an educational institution. This cluster involved four pediatric TB patients (cases H2.1, H3.1, H6, and H7), and two of them had recurrent TB episodes (cases H2.1, H2.2, H3.1, and H3.2). A heterozygous base g.819128C > T was detected in *Mtb* isolate of the case H2.1 with AF = 10%, while in H2.2, H3.2, H4, H5, H6, H7, and H8 isolates it was found with AF = 100%. Due to the very low AF of this variant in the H2.1 isolate, we hypothesize that index case and initial source case H1 was the source of infection for H2.1, and case H2.1 could be the source of infection for both H3.1 and H4. Moreover, the case H1 specimen was acquired 44.1 months earlier than the case H2.1 specimen, which indicated a long period of latent TB infection in this individual. The median-joining network, patients’ diagnoses, specimen acquisition timeline, and epidemiological data indicated, that case H2.2 was the most possible source of infection for both H3.2 and identified contacts within an educational institution (cases H5, H6, and H7).

Although cases H2.1 and H2.2 were separated by only 1 SNV, the first episode had identical *Mtb* genomes with case H4, whose specimen was acquired 19.47 months earlier than the case H2.2 specimen. In the second TB recurrence case, a 5 SNV distance was determined between H3.1 and H3.2 isolates, and the shortest path between the two nodes included a negative step followed by three positive steps. Thus, for both H2 and H3 cases, reinfection is thought to be a cause of TB recurrence.

The initial node of the cluster I median-joining network included cases I1, I2, and I4. Since only the index case I1 had positive sputum smear microscopy, we hypothesize that this TB patient was also the initial source case. The I1 case specimen was acquired 23.63 months earlier than the case I2 specimen, which indicated a long period of latent infection in at least one family member. There were two pediatric TB patients in this cluster (cases I3 and I6). Based on the small difference of 0.17 months between the acquisition of I2 and I3 specimens, and the previously mentioned sputum smear microscopy results we suggest that the index case I1 was also the source of infection for pediatric TB case I3, which supported the epidemiological data. A heterozygous base was detected in the *Mtb* isolate obtained from case I3: g.3872868G > T was found in I3 and I5 isolates with AF = 71% and AF = 100%, respectively. It indicated TB transmission from the adolescent (case I3) to the adult individual (case I5), which was also supported by positive sputum smear microscopy of case I3. Although pediatric TB case I6 was strongly epidemiologically linked only with case I3, the median-joining network revealed, that I3 could not be the source of infection as I6 was only directly connected with I3 family members I1, I2, and I4. Furthermore, a long latent TB infection period of at least 43.93 months is expected in case I6 based on the specimen acquisition timeline of this cluster.

#### 3.3.3 Sub-lineage 4.8

Among 14 *Mtb* isolates belonging to the sub-lineage 4.8, SIT53 spoligotype was determined for 11 isolates obtained from 10 patients (clusters J-L), and three isolates of three patients had SIT280 corresponding pattern (cluster M). Three pulmonary TB patients were involved in each cluster, and the average time between specimen collection from the first and last cluster TB patients was 42.22 months (range 0.23–84.13). All but one clusters were drug-susceptible, while in the cluster K *Mtb* isolates of two drug resistance types were present: cases K2 and K4 were Hr, while K1 and K3 were MDR. The genetic distance range between *Mtb* isolates belonging to three different SIT53 clusters was 110–377 SNVs, and between SIT53 and SIT280 *Mtb* isolates the distance range was 243–387 SNVs (Figure 4C). Within clusters, the SNV distances between *Mtb* isolates were 0–4 (J), 4–137 (K), 1–7 (L), and 0–1 (M).

Median-joining network topology of the clusters J and K revealed that initial source cases of both clusters remained unidentified, as both networks split creating two branches. However, the cluster J network supported the epidemiological data, which put forward the index case J1 as the source of infection for pediatric TB case J2. Although case J3 was defined as a contact of J1, the path between these cases included one negative and one positive step overturning the possibility of direct transmission events. Moreover, since the case J1 specimen was acquired 84.14 months earlier than the J3 specimen, a long period of latent infection in this individual is suspected, and multiple unidentified infectious TB cases belonging to this cluster are possible.

Based on the differing SNV analysis between cluster K isolates, we suspect that there were two unidentified initial source cases, which were infected with remotely related *Mtb* strains both belonging to the SIT53 spoligotype but having different drug resistance patterns (Hr and MDR). Notably, in the case of K3, the coinfection of both *Mtb* strains was detected, and the Hr *Mtb* strain was dominant (AF = 60–80%). Unfortunately, due to apparent unidentified active TB cases in this cluster, identification of the source of infection for the pediatric TB case K4 or transmission analysis of both *Mtb* strains could not be performed. Similarly, in cluster M, the exact source of infection for the pediatric TB case M1 could not be identified, as *Mtb* isolates of equally possible source cases M2 and M3 were identical, and patients’ specimens were acquired within a few days.

According to the cluster L median-joining network, the index case L1 was also the initial source of infection, whose specimen was obtained 23.47 months earlier than the case L2 specimen, suggesting a long latent TB infection period in this individual. As network nodes of cases L2.1 and L3 were directly linked and exhibited genetic distance of only 2 SNVs, we suggest that case L2.1 was the source of infection for pediatric TB case L3, which supported the epidemiological investigation results. One patient in this cluster had a recurrent TB episode (cases L2.1 and L2.2), and a heterozygous base was detected in *Mtb* isolate of the first episode: g.1940834C > T was found in L2.1 and L3 isolates with AF = 50% and AF = 100%, respectively. A 5 SNV distance including a heterozygous base was identified between L2.1 and L2.2 isolates, and the path between the two network nodes included a negative step followed by a positive step. However, the L2.1 *Mtb* isolate harbored an unfixed variant and no potential source of infection for the second episode could be identified due to the wide period between L1 and L2.2 specimen acquisition of 65.4 months. Thus, reactivation is a plausible recurrent TB cause in this case.

#### 3.3.4 Sub-lineage 4.2.1

Sub-lineage 4.2.1 and SIT3340 spoligotype were determined for 11 *Mtb* isolates (clusters N and O) obtained from 10 pulmonary TB patients and one tuberculous pleurisy patient (case N2). Clusters N and O involved eight and three TB patients, respectively, and the time between specimen collection from the first and last cluster TB patients was 103.3 months in cluster N, and 9.27 months in cluster O. The SNV distance between *Mtb* isolates belonging to different clusters was only 13–19 SNVs (Figure 4D), which indicated the *Mtb* strain relatedness. The first specimen of cluster O was acquired 22.33 months later than the last specimen of cluster N. As the residence places of the patients involved in these clusters lay within approximately 40-kilometer distance, we hypothesize that all TB cases could be caused by closely related *Mtb* strains, that were transmitted within this geographical area. *Mtb* isolates within clusters were separated by 0–7 SNVs (N) and 2–3 SNVs (O).

The median-joining network topology revealed the possibility of unidentified TB cases belonging to both clusters: the cluster N network split creating two branches and separating the index case N1 from others by 2–7 SNV distance, while all paths connecting cluster O cases included one negative network step overturning the possibility of direct transmission events between patients. Therefore, the source of TB infection for the pediatric TB case O2 could not be determined.

Notably, the evolutionary direction of SNV accumulation among cluster N *Mtb* isolates did not follow the specimen acquisition timeline. The case N3 specimen was obtained 90.31 months earlier than the case N8 specimen, however, the N3 isolate harbored 2 differing SNVs. Similarly, the case N2 specimen was obtained 14.71 and 16.64 months earlier than N5 and N6 specimens, respectively, but had 2 acquired SNVs. Finally, specimens of the cases N5 and N6 were obtained 21.1 and 19.27 months earlier than the case N7 specimen, respectively, but both N5 and N6 isolates had 1 differing SNV. Therefore, we suspect long latent TB infection periods in cases N5, N6, N7, and N8 as well as more unidentified active TB cases belonging to this cluster, that could be potential sources of infection for all individuals involved. There were two pediatric TB patients in this cluster (cases N2 and N8), and unfortunately, the potential sources of infection could not be determined due to previously discussed obstacles.

Heterozygous bases were called in *Mtb* isolates obtained from the cases N4 and N5: g.2244562A > C was detected in N4 and N5 isolates with AF = 42% and AF = 22%, respectively, while in N2 and N6 isolates this variant was found with AF = 100%. This finding indicated the transmission of TB infection from the case N5 to N6. Furthermore, in N4 isolate g.3139187G > T was detected with AF = 26%, and this variant connected case N4 with N3 and N8, which harbored this variant with AF = 100%. Two heterozygous bases in the N4 *Mtb* isolate highlighted the possibility of two source cases. However, since the case N4 specimen was acquired 10.27 and 12.2 months earlier than the N5 and N6 specimens, respectively, and there were unidentified active TB cases belonging to this cluster as discussed previously, neither source of infection could be identified for the case N4 based on available data.

#### 3.3.5 Sub-lineage 2.2.1

Sixteen *Mtb* isolates obtained from 15 pulmonary TB patients belonged to the sub-lineage 2.2.1 and SIT1 spoligotype (clusters P-R). *Mtb* isolates of the clusters Q and R were Hr and cluster P was drug-susceptible. The average cluster size was 5 patients (range 3–6), while the average time between specimen collection from the first and last cluster TB patients was 37.39 months (range 27.1–52.83). The genetic distance range between *Mtb* isolates belonging to different clusters was 109–119 SNVs (Figure 4E). Within the clusters, SNV distances between *Mtb* isolates were 0–3 (P), 1–5 (Q), and zero (R). All *Mtb* isolates of cluster R were identical, which interfered with the establishment of the transmission chain and infection source identification for pediatric TB case R3. However, since the index case R1 specimen was obtained 52.8 months earlier than the R2 and R3 specimens, a long period of latent TB infection is suspected in at least one of these individuals.

The initial node of the cluster P median-joining network included the index case P1, pediatric TB case P3, and cases P4.1 and P5, whose specimens were obtained within 20.14 months, and who lived in two different households. Among these TB cases, only P1 had positive sputum smear microscopy, therefore we suspect that the index case P1 was also the initial source of infection for the family members, including pediatric TB case P3, according to the specimen acquisition timeline. One patient in this cluster experienced TB recurrence (cases P4.1 and P4.2). Although recurrent TB isolates exhibited a small difference of 2 SNVs, the network nodes of two episodes were connected via an intermediate node representing case P6. Since cases P4 and P6 shared the same household and case P6 had positive sputum smear microscopy, reinfection is thought to be a cause of TB recurrence.

The network topology of cluster Q revealed the possibility of unidentified TB cases belonging to this cluster, including the initial source of infection, as the cluster network split creating two branches. The index case Q1 specimen was acquired 24.87 months earlier than the case Q2 specimen; however, Q1 isolate harbored 2 differing SNVs. Thus, we hypothesize that cases Q2 and Q4 had long latent TB infection periods. According to the epidemiological data, case Q2 was defined as a potential source of infection for pediatric TB case Q3, while the median-joining network revealed that case Q4 transmitted TB infection to Q3. Notably, in the *Mtb* isolate of case Q6 two heterozygous bases were called: g.534971C > A and g.2033223C > T were detected with AF = 22% and AF = 77%, respectively. Both variants were identified with AF = 100% in other *Mtb* isolates: the first variant was harbored by *Mtb* isolates of the cases Q3 and Q4, while the second variant was found in Q1, Q2, and Q5 isolates. This finding suggests that case Q6 acquired TB infection from two sources (cases Q2 and Q4).

### 3.4 Analysis of recently accumulated SNVs

In total, 120 differing SNVs were detected between *Mtb* isolates separated by a maximum of 19 SNVs (Supplementary material S4): 92.5% (111/120) variants were identified in the protein-coding genes, and 7.5% (9/120) were located in the intergenic regions. No SNV accumulation pattern could be identified in this *Mtb* sample collection as most variants occurred randomly in different positions across the whole *Mtb* genome. Only in three cases did two variants accumulate in the same genomic loci: p.Ser248Asn and p.Val196Ala in the locus Rv0021c were detected in *Mtb* isolates of the cases Q1 (SIT1) and C4 (SIT254), respectively; p.Leu1306Val and p.Asp172Tyr in the locus Rv0107c (*ctpl* gene) were harbored by isolates of the cases F4 (SIT254) and B2 (SIT42), respectively; p.Gly35Ser in the locus Rv0189c (*ilvD* gene) was detected in J1 and J2 (SIT53) isolates, and synonymous variant g.221163G > T in the codon Val187 was found in case O1 *Mtb* isolates. No recently acquired variants were associated with drug resistance development.

Among recently acquired SNVs, missense variants were the most prevalent (61.3%, 68/111), followed by synonymous (36.9%, 41/111) and stop variants (1.8%, 2/111). The SNV effect occurrence distribution significantly differed from the predicted distribution [χ^2^_(2, 111)_ = 59.51, df = 2, *p* < 0.001]. Standardized residuals indicated that stop mutations (standardized residual <−7) were significantly less common than expected, while missense variants occurred more frequently (standardized residuals>6). The observations provided greater support for the uneven distribution of accumulated SNV effects (*H_1_*) than for the assumption that effects occur with equal frequency (*H_0_*). Cohen’s W value of 0.73 implied a large effect magnitude, which indicated a partial matching of the results with the SNV functional effect distribution in the population.

## 4 Discussion

This study comprised TB transmission chain delineation of 18 clusters that involved 100 active TB patients and recurrent TB cause determination for nine of these patients. Contact tracing along with *Mtb* isolate spoligotyping aimed at identifying all active TB patients countrywide that could belong to these clusters continued for 17 years. The wide time frame of epidemiological surveillance allowed the thorough investigation of disease transmission in mixed adult and pediatric populations, determination of long latent TB infection periods, and description of genetic distances between numerous *Mtb* isolates belonging to different genotypes using the WGS approach. Nonetheless, only specific clusters involving culture-confirmed pediatric TB cases were presented in this study, and the analyzed dataset could not be considered representative of the total *Mtb* bacilli population circulating in Latvia.

The sex ratio imbalance in the studied adult group corresponded to the global and European TB incidence (1, 55) as there were more male than female patients. Furthermore, the median age of adult TB patients and IQR matched the European Centre for Disease Prevention and Control data, claiming that individuals from 25 to 44 years old are more often diagnosed with TB. In the pediatric population, the risk of progression from primary *Mtb* infection to active TB disease varies depending on the patient’s age. The median pediatric TB patient age in this study was 14 years, and adolescents are considered a high-risk group as 10–20% of these patients develop active TB, while the lifetime risk is defined as 5–10% for immunocompetent adult individuals and adult-type cavitary disease is common in this population (56–58). Furthermore, children are at higher risk of getting infected with *Mtb* after household exposure rather than community exposure (59, 60), which is also reflected in our study. Among 29 pediatric TB cases, family members of 24 children living in either the same or different households were involved in the same TB cluster.

Previously conducted spoligotyping emphasized the wide *Mtb* genotype variability among studied TB clusters. Moreover, *Mtb* isolates of 13 individuals initially clustered with other TB patients based on the established epidemiological links were withdrawn from the study as they belonged to different spoligotypes than the clustered *Mtb* isolates. It reflected the scale of distinct *Mtb* genotypes’ distribution within limited geographical areas in Latvia. Herein, we also applied WGS-based strain genotyping to complement spoligotyping results. According to the SITVIT2 database (61) and recently conducted epidemiological TB studies (38–40), all five sub-lineages and eight spoligotypes in our study had been reported previously in Latvia, while drug-resistant strains belonged to the spoligotypes frequently associated with drug resistance (i.e., SIT1, SIT42, SIT50, SIT53, and SIT283). Spoligotypes belonging to Beijing (SIT1), LAM (SIT42 and SIT254), and T (SIT53) families were the most abundant in the previous studies, thus such cluster detection is not unusual.

Although the Beijing genotype has been tightly associated with increased transmissibility and unfavorable treatment outcomes (62, 63), in this study, it was determined only for three clusters involving a maximum of six epidemiologically closely linked TB patients per cluster, whereas *Mtb* isolates of the two largest clusters of 12 and 13 TB patients, which included both family members and community contacts, belonged to the LAM genotype. Furthermore, five of seven patients who experienced extrapulmonary TB had been infected with LAM *Mtb* strains. *Mtb* isolates belonging to the LAM family had been acquired from extrapulmonary TB patients before, however, this *Mtb* genotype was not predominant in studied populations (64, 65). On the contrary, each studied TB cluster of the T genotype involved only three patients, while isolates of three clusters belonged to spoligotypes that are not as widespread in Latvia (i.e., SIT3340 (two clusters) and SIT280). Further detailed studies on differences in virulence, TB disease severity, transmissibility, as well as microbial tissue tropism between *Mtb* genotypes are needed to complement existing knowledge and improve TB monitoring strategies.

*Mtb* strain genetic variability was assessed not only within TB clusters but also between all *Mtb* isolates belonging to the same sub-lineage by creating median-joining networks based on WGS data. The genetic distances between *Mtb* isolates of sub-lineages 2.2.1 and 4.3.3 were highly similar (reached 119 and 112 SNVs, respectively); however, all TB clusters of the sub-lineage 2.2.1 were located in Riga or Riga region, but sub-lineage 4.3.3 clusters occurred countrywide. On the other hand, clusters belonging to the sub-lineages 4.1.2.1 and 4.8 also covered a wide geographical area, and the genetic distances between *Mtb* isolates were significantly greater (reached 215 and 387 SNVs, respectively). This data suggests greater genetic relatedness of isolates within the sub-lineage 4.3.3 than sub-lineages 4.1.2.1 and 4.8 in Latvia which supports the results of previously performed phylogenetic analysis (40). Notably, small genetic distances of a maximum of 16 and 19 SNVs were detected between *Mtb* isolates of three sub-lineage 4.3.3 clusters and two sub-lineage 4.2.1 clusters, respectively. As *Mtb* strains belonging to the sub-lineage 4.2.1 were transmitted within approximately a 40-kilometer area, sub-lineage 4.3.3 *Mtb* strains were spread within approximately a 400-kilometer area, including the capital Riga. This finding highlights the transmission of closely related *Mtb* strains in a low-moderate-TB-incidence country within geographical areas of variable size, and the necessity of countrywide TB cluster identification to assess the transmission patterns and *Mtb* strain genetic diversity thoroughly.

Construction of TB cluster median-joining networks and differing SNV analysis between *Mtb* isolates using acquired WGS data significantly complemented previously conducted epidemiological investigation and spoligotyping results. Except for two cases, when patients involved in the genotype-matched TB cluster got infected with remotely related *Mtb* strains as indicated by WGS results, the maximum genetic distance within clusters varied between 5 and 11 SNVs, which corresponds to the commonly applied 12 SNV-threshold for epidemiologically relevant transmission cluster identification, which was also used in this study. Furthermore, none of the directly connected TB cases in the network exhibited a distance of more than 4 SNVs, when 5 SNV-distance is a widely accepted threshold for inferring recent transmission events between TB patients (19, 20, 27, 28, 66). Among 120 detected differing SNVs between closely related *Mtb* isolates, no drug-resistance-associated variants were detected, which indicated that no resistance-triggering factors were present in this study.

However, several drawbacks of applying the SNV distance threshold have been reported previously. Firstly, these thresholds were mostly determined in low-incidence settings and could not always be applied for TB transmission analysis in high-burden areas with highly clonal *Mtb* strain populations when a small SNV distance between TB patients’ isolates does not necessarily indicate their involvement in the same transmission chain (20, 21, 67). Secondly, the enhanced intra-patient *Mtb* microevolution might affect the genetic distances between isolates exceeding the proposed thresholds (68), while the long periods of latent TB infection might cause significant delays in the precise delineation of transmission chains (69). Thirdly, although the application of SNV distance thresholds provided valuable insights into TB transmission dynamics in different settings and helped to rule out those cases when TB transmission was unlikely (19, 70), the direction and timing of specific person-to-person transmission events mostly remained unclear (71, 72). Several computational tools have been developed to infer individual transmission events by combining pathogen genomic data with underlying epidemiological models which have been applied to *Mtb* WGS data in multiple TB transmission studies (67, 72–78). Nonetheless, generated transmission trees still should be interpreted critically by considering available clinical (diagnosis, specimen collection date, sputum smear microscopy results) and epidemiological (geospatial analysis and contact tracing) data regarding studied clusters (74, 75). Therefore, at this point, the development of a comprehensive algorithm allowing to precisely delineate TB transmission chains is still essential.

Low mycobacterial mutation rate previously defined as 0.3–0.5 SNVs/genome/year in several transmission studies (19, 67, 79) can vary depending on multiple mycobacteria- (strain genotype and drug resistance), host- and therapy-related factors (patient adherence and treatment regimen) (25, 79, 80). Indeed, most of the studied *Mtb* isolates demonstrated low genetic variability with the presence of identical isolates in 13 clusters, which limited the precision of complete transmission chain delineating. Furthermore, in cases of some identical *Mtb* isolates, patients’ specimens were collected within a few days, complicating the performed analysis even more. Some of these cases were solved by including heterozygous bases in the differing SNV analysis. Heterozygous variants reflect an incomplete SNV accumulation, which could potentially indicate the transmission direction within a cluster (81). In this study, 11 heterozygous bases were detected in *Mtb* isolates with AF = 10–73%. In four cases (clusters F, I, H, and N), it helped to identify the putative direct transmission events between patients, and in three cases (clusters A, C, and D), heterozygous bases were harbored by the cluster index cases, indicating that these patients also were initial source cases. Moreover, in three isolates the presence of multiple heterogenous bases indicated, that the patient got infected either with two remotely related *Mtb* strains (case K3) or from two other TB patients involved in the same cluster (cases N4 and Q7). As, according to the patients’ specimen acquisition dates, heterozygous bases were detected in *Mtb* isolates that were obtained earlier than isolates harboring the same variant with homozygous frequency, the heterozygous base identification step improved the discriminative power of the median-joining network analysis.

As the main objective of epidemiological investigation and conventional genotyping was defining the source of TB infection for pediatric patients, we assessed if WGS data could support the initial assumptions. During this analysis, we also considered patients’ diagnoses, sputum smear microscopy results, and specimen acquisition dates. Unfortunately, sources of infection could be precisely determined only for 11 of 29 pediatric TB patients either supporting the epidemiological data (cases B1, B2, C2, F3, H2.1, H3.1, J2, L3, and P3) or defining a different source case (cases A8 and Q3). In other cases, previously mentioned low *Mtb* genetic variability (cases D4, E2, E3, E4, E7, E9, G4, H6, H7, I3, I6, M1, and R3) interfered with precise source case determination, while unidentified active TB cases belonging to the cluster (cases F1, K4, and O2) and accumulated SNVs occurring inversely to specimen acquisition timeline (cases N2 and N8) made source case detection based on WGS data impossible. The latter-mentioned finding also interfered with transmission chain delineation in clusters G, N, and Q, as *Mtb* isolates which were acquired earlier in the study period (cases G1, N2, N3, N4, N5, N6, and Q1) harbored additional SNVs in comparison with directly connected isolates obtained 16.07–55.87 months later, indicating long periods of latent TB infection in those patients. In nine other clusters (A, C, D, E, H, I, J, L, and R), putative latent TB infection periods of up to 123.27 months were identified in several patients, and their isolates acquired limited genetic distance of 0–1 SNV compared to possible sources of infection. Currently, available data on mycobacterial mutation rate during the latency period are controversial: some studies report, that *Mtb* accumulates new variants at a similar rate while being dormant (82, 83), and other studies imply that mutagenesis during latent infection is significantly slower than in the active state (84, 85). Therefore, we hypothesize that our data could indicate either a slower mutation rate of dormant mycobacteria or a faster rate during *Mtb* strain transmission between individuals; however, the mycobacterial mutation rate in both dormant and active states should be studied further.

Finally, we also determined the causes of recurrent TB episodes for nine patients, who were involved in studied clusters. The construction of median-joining networks including all known epidemiologically linked and genotype-matched TB cases along with analysis of available clinical, epidemiological, and geospatial data allowed more precise TB recurrence cause prediction in several recently conducted studies (32–34). These approaches significantly complement the commonly applied distinguishing algorithm based on SNV-distance identification between *Mtb* isolates that represent each active episode of recurrent TB patient allowing the determination of reinfection with clonal or closely related *Mtb* strain. Herein, excluding the apparent reinfection case in cluster C with 97 SNV distance between TB episodes, *Mtb* isolates obtained from the first and second episodes of eight patients were separated by a maximum of 5 SNVs. Based on the genetic distance thresholds of 5–12 SNVs proposed in several studies (23–25, 29, 30), these cases would be classified as endogenous reactivations. However, only in two cases (clusters G and L) no potential sources of infection for the second episode could be identified based on the available epidemiological and geographical data, specimen acquisition timeline of the cluster, as well as network distances and topology. In the remaining six cases at least one potential source of infection for recurrent TB patients could be identified within the cluster, therefore reinfection was a more reliable recurrence cause.

In our previous study (40), we distinguished endogenous reactivation from exogenous reinfection by detecting differing SNVs between *Mtb* isolates acquired from the first and second TB episodes, and only those cases, when the first isolate harbored zero differing SNVs, were classified as “possible reactivation.” Nevertheless, in this study, only one of six recurrent TB patients, who according to our investigation results experienced reinfection with closely related *Mtb* strain, harbored 1 differing SNV in the *Mtb* isolate of the first episode. Thus, the identification of differing SNVs between recurrent TB patient isolates cannot replace the complex approach applied in this study, and WGS data analysis of all epidemiologically linked patients’ *Mtb* isolates is required for more accurate recurrence cause determination.

To summarize, WGS-data-based median-joining networks of studied TB clusters significantly complemented epidemiological investigation and *Mtb* strain spoligotyping results, when analyzed considering relevant clinical data such as patients’ diagnoses, sputum smear microscopy results, and specimen acquisition date. Overall, this comprehensive approach allowed the evaluation of the known index case as the initial source case and enabled the identification of putative infection sources for patients of interest, herein—pediatric TB patients. Moreover, it facilitated a more precise determination of recurrent TB episode causes when the genetic distance between *Mtb* isolates was ≤5 SNVs. Unidentified active TB cases belonging to the cluster, variable *Mtb* mutation rate in active and dormant states, tight specimen collection date timeline of genetically identical *Mtb* isolates, and low *Mtb* genetic variability interfered with transmission chain delineation in this study, while the inclusion of heterozygous SNVs in differing variant analysis between cluster *Mtb* isolates assisted in the identification of direct transmission events. In our opinion, the applied cluster investigation approach could be implemented as a part of a local TB surveillance program if WGS of all acquired *Mtb* isolates is routinely performed. However, it would require substantial resources to conduct such an investigation countrywide even in a low-burden setting.

## Data availability statement

The datasets presented in this study can be found in online repositories. The names of the repository/repositories and accession number(s) can be found at: https://www.ebi.ac.uk/ena, PRJEB71941.

## Ethics statement

The studies involving humans were approved by the Riga Stradiņš University Research Ethics Committee (Nr. 6-1/06/12; 28.05.2020), the Centre for Disease Prevention and Control of Latvia (Nr.14; 22.07.2020), and the Science Department of Riga East University Hospital (Nr. ZD/08-06/01-20/215; 10.09.2020). The studies were conducted in accordance with the local legislation and institutional requirements. The ethics committee/institutional review board waived the requirement of written informed consent for participation from the participants or the participants’ legal guardians/next of kin because of the retrospective nature of this study as only previously acquired mycobacterial DNA samples and patients’ medical records were investigated in this study.

## Author contributions

DS: Conceptualization, Data curation, Formal analysis, Investigation, Methodology, Software, Validation, Visualization, Writing – original draft. IO: Conceptualization, Data curation, Investigation, Methodology, Validation, Writing – original draft, Writing – review & editing. IP: Conceptualization, Data curation, Investigation, Resources, Writing – review & editing. JĶ: Software, Writing – review & editing. AVa: Investigation, Writing – original draft. AVī: Investigation, Writing – review & editing. IN: Resources, Writing – review & editing. IB: Investigation, Writing – review & editing. VU: Investigation, Writing – review & editing. VČ: Investigation, Writing – review & editing. DB: Supervision, Writing – review & editing. RR: Conceptualization, Funding acquisition, Methodology, Project administration, Resources, Supervision, Validation, Writing – review & editing.

## References

[1] World Health Organization. Global tuberculosis report 2023. Geneva: World Health Organization (2023) Licence: CC BY-NC-SA 3.0 IGO.

[2] World Health Organization. Implementing the end TB strategy: The essentials, 2022 update. Geneva: World Health Organization (2022) Licence: CC BY-NC-SA 3.0 IGO.

[3] Kendall EA Fofana MO Dowdy DW. Burden of transmitted multidrug resistance in epidemics of tuberculosis: a transmission modelling analysis. Lancet Respir Med. (2015) 3:963–72. doi: 10.1016/S2213-2600(15)00458-0, PMID: 26597127 PMC4684734

[4] Erkens CGM Kamphorst M Abubakar I Bothamley GH Chemtob D Haas W. Tuberculosis contact investigation in low prevalence countries: a European consensus. Eur Respir J. (2010) 36:925–49. doi: 10.1183/09031936.00201609, PMID: 20889463

[5] Fox GJ Barry SE Britton WJ Marks GB. Contact investigation for tuberculosis: a systematic review and meta-analysis. Eur Respir J. (2013) 41:140–56. doi: 10.1183/09031936.00070812, PMID: 22936710 PMC3533588

[6] World Health Organization. Guidance for national tuberculosis programmes on the management of tuberculosis in children. 2nd ed. Geneva: World Health Organization (2023) Licence: CC BY-NC-SA 3.0 IGO. 24999516

[7] Starke JR Erkens C Ritz N Kitai I. Strengthening Tuberculosis Services for Children and Adolescents in low endemic settings. Pathogens. (2022) 11:158. doi: 10.3390/pathogens11020158, PMID: 35215101 PMC8877840

[8] Brooks-Pollock E Danon L Altes HK Davidson JA Pollock AMT Van Soolingen D. A model of tuberculosis clustering in low incidence countries reveals more transmission in the United Kingdom than the Netherlands between 2010 and 2015. PLoS Comput Biol. (2020) 16:e1007687. doi: 10.1371/journal.pcbi.1007687, PMID: 32218567 PMC7141699

[9] Chaoui I Zozio T Lahlou O Sabouni R Abid M El Aouad R. Contribution of spoligotyping and MIRU-VNTRs to characterize prevalent *Mycobacterium tuberculosis* genotypes infecting tuberculosis patients in Morocco. Infect Genet Evol. (2014) 21:463–71. doi: 10.1016/j.meegid.2013.05.023 23732366

[10] Fandinho FC Kritski AL Hofer C Júnior Conde H Ferreira RM Saad MH. RFLP patterns and risk factors for recent tuberculosis transmission among hospitalized tuberculosis patients in Rio de Janeiro, Brazil. Trans R Soc Trop Med Hyg. (2000) 94:271–5. doi: 10.1016/s0035-9203(00)90317-1, PMID: 10974996

[11] Antusheva E Mironuk O Tarasova I Eliseev P Plusnina G Ridell M. Outbreak of tuberculosis in a closed setting: views on transmission based on results from molecular and conventional methods. J Hosp Infect. (2016) 93:187–90. doi: 10.1016/j.jhin.2016.02.015, PMID: 27105749

[12] Ghebremichael S Petersson R Koivula T Pennhag A Romanus V Berggren I. Molecular epidemiology of drug-resistant tuberculosis in Sweden. Microbes Infect. (2008) 10:699–705. doi: 10.1016/j.micinf.2008.03.006 18485780

[13] Roetzer A Diel R Kohl TA Rückert C Nübel U Blom J. Whole genome sequencing versus traditional genotyping for investigation of a *Mycobacterium tuberculosis* outbreak: a longitudinal molecular epidemiological study. PLoS Med. (2013) 10:e1001387. doi: 10.1371/journal.pmed.1001387, PMID: 23424287 PMC3570532

[14] Warren RM Streicher EM Charalambous S Churchyard G Van Der Spuy GD Grant AD. Use of spoligotyping for accurate classification of recurrent tuberculosis. J Clin Microbiol. (2002) 40:3851–3. doi: 10.1128/JCM.40.10.3851-3853.2002, PMID: 12354898 PMC130897

[15] Dobler CC Marks GB Simpson SE Crawford ABH. Recurrence of tuberculosis at a Sydney chest clinic between 1994 and 2006: reactivation or reinfection? Med J Aust. (2008) 188:153–5. doi: 10.5694/j.1326-5377.2008.tb01558.x 18241171

[16] Zong Z Huo F Shi J Jing W Ma Y Liang Q. Relapse versus reinfection of recurrent Tuberculosis patients in a National Tuberculosis Specialized Hospital in Beijing, China. Front Microbiol. (2018) 9:1858. doi: 10.3389/fmicb.2018.01858, PMID: 30154770 PMC6102324

[17] Luzze H Johnson DF Dickman K Mayanja-Kizza H Okwera A Eisenach K. Relapse more common than reinfection in recurrent tuberculosis 1-2 years post treatment in urban Uganda. Int J Tuberc Lung Dis. (2013) 17:361–7. doi: 10.5588/ijtld.11.0692, PMID: 23407224 PMC6623981

[18] McIvor A Koornhof H Kana BD. Relapse, re-infection and mixed infections in tuberculosis disease. Pathog Dis. (2017) 75:75. doi: 10.1093/femspd/ftx020, PMID: 28334088

[19] Walker TM Ip CLC Harrell RH Evans JT Kapatai G Dedicoat MJ. Whole-genome sequencing to delineate *Mycobacterium tuberculosis* outbreaks: a retrospective observational study. Lancet Infect Dis. (2013) 13:137–46. doi: 10.1016/S1473-3099(12)70277-3, PMID: 23158499 PMC3556524

[20] Dixit A Freschi L Vargas R Calderon R Sacchettini J Drobniewski F. Whole genome sequencing identifies bacterial factors affecting transmission of multidrug-resistant tuberculosis in a high-prevalence setting. Sci Rep. (2019) 9:5602. doi: 10.1038/s41598-019-41967-8, PMID: 30944370 PMC6447560

[21] Bjorn-Mortensen K Soborg B Koch A Ladefoged K Merker M Lillebaek T. Tracing *Mycobacterium tuberculosis* transmission by whole genome sequencing in a high incidence setting: a retrospective population-based study in East Greenland. Sci Rep. (2016) 6:33180. doi: 10.1038/srep33180, PMID: 27615360 PMC5018808

[22] Wyllie DH Davidson JA Grace Smith E Rathod P Crook DW Peto TEA. A quantitative evaluation of MIRU-VNTR typing against whole-genome sequencing for identifying *Mycobacterium tuberculosis* transmission: a prospective observational cohort study. EBioMedicine. (2018) 34:122–30. doi: 10.1016/j.ebiom.2018.07.019, PMID: 30077721 PMC6116353

[23] Bryant JM Harris SR Parkhill J Dawson R Diacon AH van Helden P. Whole-genome sequencing to establish relapse or re-infection with *Mycobacterium tuberculosis*: a retrospective observational study. Lancet Respir Med. (2013) 1:786–92. doi: 10.1016/S2213-2600(13)70231-5, PMID: 24461758 PMC3861685

[24] Parvaresh L Crighton T Martinez E Bustamante A Chen S Sintchenko V. Recurrence of tuberculosis in a low-incidence setting: a retrospective cross-sectional study augmented by whole genome sequencing. BMC Infect Dis. (2018) 18:265. doi: 10.1186/s12879-018-3164-z, PMID: 29879906 PMC5992641

[25] Korhonen V Smit PW Haanperä M Casali N Ruutu P Vasankari T. Whole genome analysis of *Mycobacterium tuberculosis* isolates from recurrent episodes of tuberculosis, Finland, 1995–2013. Clin Microbiol Infect. (2016) 22:549–54. doi: 10.1016/j.cmi.2016.03.014, PMID: 27021423

[26] Hatherell HA Colijn C Stagg HR Jackson C Winter JR Abubakar I. Interpreting whole genome sequencing for investigating tuberculosis transmission: a systematic review. BMC Med. (2016) 14:21. doi: 10.1186/s12916-016-0566-x, PMID: 27005433 PMC4804562

[27] Ji L Tao FX Yu YF Liu JH Yu FH Bai CL. Whole-genome sequencing to characterize the genetic structure and transmission risk of *Mycobacterium tuberculosis* in Yichang city of China. Front Public Health. (2023) 10:1047965. doi: 10.3389/fpubh.2022.1047965, PMID: 36699912 PMC9868839

[28] Lin Y Du Y Shen H Guo Y Wang T Lai K. Transmission of *Mycobacterium tuberculosis* in schools: a molecular epidemiological study using whole-genome sequencing in Guangzhou, China. Front Public Health. (2023) 11:1156930. doi: 10.3389/fpubh.2023.1156930, PMID: 37250072 PMC10219607

[29] Witney AA Bateson ALE Jindani A Phillips PPJ Coleman D Stoker NG. Use of whole-genome sequencing to distinguish relapse from reinfection in a completed tuberculosis clinical trial. BMC Med. (2017) 15:71. doi: 10.1186/s12916-017-0834-4, PMID: 28351427 PMC5371199

[30] Guerra-Assuncąõ JA Houben RMGJ Crampin AC Mzembe T Mallard K Coll F. Recurrence due to relapse or reinfection with *Mycobacterium tuberculosis*: a whole-genome sequencing approach in a large, population-based cohort with a high HIV infection prevalence and active follow-up. J Infect Dis. (2015) 211:1154–63. doi: 10.1093/infdis/jiu574, PMID: 25336729 PMC4354982

[31] Dippenaar A De Vos M Marx FM Adroub SA van Helden PD Pain A. Whole genome sequencing provides additional insights into recurrent tuberculosis classified as endogenous reactivation by IS6110 DNA fingerprinting. Infect Genet Evol. (2019) 75:103948. doi: 10.1016/j.meegid.2019.103948, PMID: 31276801

[32] Pérez-Lago L Monteserin J Paul R Maus SR Yokobori N Herranz M. Recurrences of multidrug-resistant tuberculosis: strains involved, within-host diversity, and fine-tuned allocation of reinfections. Transbound Emerg Dis. (2022) 69:327–36. doi: 10.1111/tbed.13982 33411991

[33] Folkvardsen DB Norman A Rasmussen EM Lillebaek T Jelsbak L Andersen ÅB. Recurrent tuberculosis in patients infected with the predominant *Mycobacterium tuberculosis* outbreak strain in Denmark. New insights gained through whole genome sequencing. Infect Genet Evol. (2020) 80:104169. doi: 10.1016/j.meegid.2020.104169, PMID: 31918042

[34] Wollenberg K Harris M Gabrielian A Ciobanu N Chesov D Long A. A retrospective genomic analysis of drug-resistant strains of *M. tuberculosis* in a high-burden setting, with an emphasis on comparative diagnostics and reactivation and reinfection status. BMC Infect Dis. (2020) 20:17. doi: 10.1186/s12879-019-4739-z, PMID: 31910804 PMC6947865

[35] World Health Organization. WHO global lists of high burden countries for tuberculosis (TB) In: TB/HIV and multidrug/rifampicin-resistant TB (MDR/RR-TB), 2021–2025. Geneva: World Health Organization (2021) Licence: CC BY-NC-SA 3.0 IGO

[36] World Health Organization. Tuberculosis profile: Latvia.(2022) Available at: https://worldhealthorg.shinyapps.io/tb_profiles/?_inputs_&entity_type=%22country%22&iso2=%22LV%22&lan=%22EN%22.

[37] Kuksa L Riekstina V Leimane V Ozere I Skenders G Vandenbergh R. Multi- and extensively drug-resistant tuberculosis in Latvia: trends, characteristics and treatment outcomes. Public Health Action. (2014) 4:47–53. doi: 10.5588/pha.14.0041, PMID: 26393098 PMC4547513

[38] Vīksna A Sadovska D Berge I Bogdanova I Vaivode A Freimane L. Genotypic and phenotypic comparison of drug resistance profiles of clinical multidrug-resistant *Mycobacterium tuberculosis* isolates using whole genome sequencing in Latvia. BMC Infect Dis. (2023) 23:638. doi: 10.1186/s12879-023-08629-7, PMID: 37770850 PMC10540372

[39] Pole I Trofimova J Norvaisa I Supply P Skenders G Nodieva A. Analysis of *Mycobacterium tuberculosis* genetic lineages circulating in Riga and Riga region, Latvia, isolated between 2008 and 2012. Infect Genet Evol. (2020) 78:104126. doi: 10.1016/j.meegid.2019.104126, PMID: 31783188

[40] Sadovska D Nodieva A Pole I Ķimsis J Vīksna A Ozere I. Advantages of analysing both pairwise SNV-distance and differing SNVs between *Mycobacterium tuberculosis* isolates for recurrent tuberculosis cause determination. Microb Genom. (2023) 9:mgen000956. doi: 10.1099/mgen.0.000956, PMID: 36951900 PMC10132068

[41] Strouse PJ Trout AT Offiah AC. Editors’ notebook: what is ‘pediatric’? Pediatr Radiol. (2022) 52:2241–2. doi: 10.1007/s00247-022-05484-7, PMID: 36018347 PMC9411830

[42] van Soolingen D Hermans PWM de Haas PEW Soll DR van Embden JDA. Occurrence and stability of insertion sequences in *Mycobacterium tuberculosis* complex strains: evaluation of an insertion sequence-dependent DNA polymorphism as a tool in the epidemiology of tuberculosis. J Clin Microbiol. (1991) 29:2578–86. doi: 10.1128/jcm.29.11.2578-2586.1991, PMID: 1685494 PMC270376

[43] Kamerbeek J Schouls L Kolk A van Agterveld M van Soolingen D Kuijper S. Simultaneous detection and strain differentiation of *Mycobacterium tuberculosis* for diagnosis and epidemiology. J Clin Microbiol. (1997) 35:907–14. doi: 10.1128/jcm.35.4.907-914.1997 9157152 PMC229700

[44] Afgan E Baker D Batut B Van Den Beek M Bouvier D Ech M. The galaxy platform for accessible, reproducible and collaborative biomedical analyses: 2018 update. Nucleic Acids Res. (2018) 46:W537–44. doi: 10.1093/nar/gky379, PMID: 29790989 PMC6030816

[45] Comas Ĩ Chakravartti J Small PM Galagan J Niemann S Kremer K. Human T cell epitopes of *Mycobacterium tuberculosis* are evolutionarily hyperconserved. Nat Genet. (2010) 42:498–503. doi: 10.1038/ng.590, PMID: 20495566 PMC2883744

[46] Phelan JE O’Sullivan DM Machado D Ramos J Oppong YEA Campino S. Integrating informatics tools and portable sequencing technology for rapid detection of resistance to anti-tuberculous drugs. Genome Med. (2019) 11:41. doi: 10.1186/s13073-019-0650-x, PMID: 31234910 PMC6591855

[47] World Health Organization. Catalogue of mutations in *Mycobacterium tuberculosis* complex and their association with drug resistance. Geneva: World Health Organization (2021) Licence: CC BY-NC-SA 3.0 IGO.

[48] Hoang DT Chernomor O von Haeseler A Minh BQ Vinh LS. UFBoot2: improving the ultrafast bootstrap approximation. Mol Biol Evol. (2018) 35:518–22. doi: 10.1093/molbev/msx281, PMID: 29077904 PMC5850222

[49] Kalyaanamoorthy S Minh BQ Wong TKF von Haeseler A Jermiin LS. ModelFinder: fast model selection for accurate phylogenetic estimates. Nat Methods. (2017) 14:587–9. doi: 10.1038/nmeth.4285, PMID: 28481363 PMC5453245

[50] Minh BQ Schmidt HA Chernomor O Schrempf D Woodhams MD von Haeseler A. IQ-TREE 2: new models and efficient methods for phylogenetic inference in the genomic era. Mol Biol Evol. (2020) 37:1530–4. doi: 10.1093/molbev/msaa015, PMID: 32011700 PMC7182206

[51] Bandelt HJ Forster P Röhl A. Median-joining networks for inferring intraspecific phylogenies. Mol Biol Evol. (1999) 16:37–48. doi: 10.1093/oxfordjournals.molbev.a026036, PMID: 10331250

[52] Mathema B Andrews JR Cohen T Borgdorff MW Behr M Glynn JR. Drivers of Tuberculosis transmission. J Infect Dis. (2017) 216:S644–53. doi: 10.1093/infdis/jix354, PMID: 29112745 PMC5853844

[53] World Health Organization. WHO consolidated guidelines on tuberculosis. Module 4: Treatment - drug-resistant tuberculosis treatment, 2022 update. Geneva: World Health Organization (2022) Licence: CC BY-NC-SA 3.0 IGO. 36630546

[54] World Health Organization. Meeting report of the WHO expert consultation on the definition of extensively drug-resistant tuberculosis, 27–29 October 2020. Geneva: World Health Organization (2021) Licence: CC BY-NC-SA 3.0 IGO.

[55] European Centre for Disease Prevention and Control. Tuberculosis In: ECDC. Annual epidemiological report for 2021. Stockholm: ECDC (2023)

[56] Perez-Velez CM Marais BJ. Tuberculosis in children. N Engl J Med. (2012) 367:348–61. doi: 10.1056/NEJMra1008049 22830465

[57] Baguma R Mbandi SK Rodo MJ Erasmus M Day J Makhethe L. Inflammatory determinants of differential Tuberculosis risk in pre-adolescent children and young adults. Front Immunol. (2021) 12:639965. doi: 10.3389/fimmu.2021.639965, PMID: 33717192 PMC7947716

[58] Cruz AT Starke JR. Clinical manifestations of tuberculosis in children. Paediatr Respir Rev. (2007) 8:107–17. doi: 10.1016/j.prrv.2007.04.008 17574154

[59] Martinez L Shen Y Mupere E Kizza A Hill PC Whalen CC. Transmission of *Mycobacterium tuberculosis* in households and the community: a systematic review and Meta-analysis. Am J Epidemiol. (2017) 185:1327–39. doi: 10.1093/aje/kwx025, PMID: 28982226 PMC6248487

[60] Sun L Qi X Guo Y Qi H Li J Wu X. Tuberculosis infection screening in children with close contact: a hospital-based study. BMC Infect Dis. (2021) 21:815. doi: 10.1186/s12879-021-06480-2, PMID: 34388985 PMC8364055

[61] Couvin D David A Zozio T Rastogi N. Macro-geographical specificities of the prevailing tuberculosis epidemic as seen through SITVIT2, an updated version of the *Mycobacterium tuberculosis* genotyping database. Infect Genet Evol. (2019) 72:31–43. doi: 10.1016/j.meegid.2018.12.030, PMID: 30593925

[62] Karmakar M Trauer JM Ascher DB Denholm JT. Hyper transmission of Beijing lineage *Mycobacterium tuberculosis*: systematic review and meta-analysis. J Infect. (2019) 79:572–81. doi: 10.1016/j.jinf.2019.09.016, PMID: 31585190

[63] Liu Q Wang D Martinez L Lu P Zhu L Lu W. *Mycobacterium tuberculosis* Beijing genotype strains and unfavourable treatment outcomes: a systematic review and meta-analysis. Clin Microbiol Infect. (2020) 26:180–8. doi: 10.1016/j.cmi.2019.07.016 31336202

[64] Uddin MKM Ather MF Rahman A Nasrin R Rahman SMM Kabir S. Genetic diversity and characterization of *M. tuberculosis* isolates causing extrapulmonary tuberculosis in Bangladesh. Infect Genet Evol. (2021) 95:105052. doi: 10.1016/j.meegid.2021.105052, PMID: 34454121

[65] Tadesse M Abebe G Bekele A Bezabih M De Rijk P Meehan J. The predominance of Ethiopian specific *Mycobacterium tuberculosis* families and minimal contribution of *Mycobacterium bovis* in tuberculous lymphadenitis patients in Southwest Ethiopia. Infect Genet Evol. (2017) 55:251–9. doi: 10.1016/j.meegid.2017.09.016, PMID: 28919549

[66] Shibabaw A Gelaw B Ghanem M Legall N Schooley AM Soehnlen MK. Molecular epidemiology and transmission dynamics of multi-drug resistant tuberculosis strains using whole genome sequencing in the Amhara region, Ethiopia. BMC Genomics. (2023) 24:400. doi: 10.1186/s12864-023-09502-2, PMID: 37460951 PMC10351181

[67] Guerra-Assunção JA Crampin AC Houben RM Mzembe T Mallard K Coll F. Large-scale whole genome sequencing of *M. tuberculosis* provides insights into transmission in a high prevalence area. eLife. (2015) 4:e05166. doi: 10.7554/eLife.05166, PMID: 25732036 PMC4384740

[68] Pérez-Lago L Comas I Navarro Y González-Candelas F Herranz M Bouza E. Whole genome sequencing analysis of intrapatient microevolution in *Mycobacterium tuberculosis*: potential impact on the inference of tuberculosis transmission. J Infect Dis. (2014) 209:98–108. doi: 10.1093/infdis/jit439, PMID: 23945373

[69] Nelson KN Talarico S Poonja S McDaniel CJ Cilnis M Chang AH. Mutation of *mycobacterium tuberculosis* and implications for using whole-genome sequencing for investigating recent Tuberculosis transmission. Front Public Health. (2022) 9:790544. doi: 10.3389/fpubh.2021.790544, PMID: 35096744 PMC8793027

[70] Luo T Yang C Peng Y Lu L Sun G Wu J. Whole-genome sequencing to detect recent transmission of *Mycobacterium tuberculosis* in settings with a high burden of tuberculosis. Tuberculosis (Edinb). (2014) 94:434–40. doi: 10.1016/j.tube.2014.04.005, PMID: 24888866 PMC4409578

[71] Stimson J Gardy J Mathema B Crudu V Cohen T Colijn C. Beyond the SNP threshold: identifying outbreak clusters using inferred transmissions. Mol Biol Evol. (2019) 36:587–603. doi: 10.1093/molbev/msy242, PMID: 30690464 PMC6389316

[72] Didelot X Gardy J Colijn C. Bayesian inference of infectious disease transmission from whole-genome sequence data. Mol Biol Evol. (2014) 31:1869–79. doi: 10.1093/molbev/msu121, PMID: 24714079 PMC4069612

[73] Yang C Lu L Warren JL Wu J Jiang Q Zuo T. Internal migration and transmission dynamics of tuberculosis in Shanghai, China: an epidemiological, spatial, genomic analysis. Lancet Infect Dis. (2018) 18:788–95. doi: 10.1016/S1473-3099(18)30218-4, PMID: 29681517 PMC6035060

[74] Ayabina D Ronning JO Alfsnes K Debech N Brynildsrud OB Arnesen T. Genome-based transmission modelling separates imported tuberculosis from recent transmission within an immigrant population. Microb Genom. (2018) 4:e000219. doi: 10.1099/mgen.0.000219, PMID: 30216147 PMC6249437

[75] Sobkowiak B Romanowski K Sekirov I Gardy JL Johnston JC. Comparing *Mycobacterium tuberculosis* transmission reconstruction models from whole genome sequence data. Epidemiol Infect. (2023) 151:e105. doi: 10.1017/S0950268823000900, PMID: 37293984 PMC10369424

[76] Didelot X Fraser C Gardy J Colijn C. Genomic infectious disease epidemiology in partially sampled and ongoing outbreaks. Mol Biol Evol. (2017) 34:997–1007. doi: 10.1093/molbev/msw275, PMID: 28100788 PMC5850352

[77] Sobkowiak B Banda L Mzembe T Crampin AC Glynn JR Clark TG. Bayesian reconstruction of *Mycobacterium tuberculosis* transmission networks in a high incidence area over two decades in Malawi reveals associated risk factors and genomic variants. Microb Genom. (2020) 6:e000361. doi: 10.1099/mgen.0.000361, PMID: 32234123 PMC7276699

[78] Séraphin MN Didelot X Nolan DJ May JR Khan MSR Murray ER. Genomic investigation of a *Mycobacterium tuberculosis* outbreak involving prison and community cases in Florida, United States. Am J Trop Med Hyg. (2018) 99:867–74. doi: 10.4269/ajtmh.17-0700, PMID: 29987998 PMC6159577

[79] Bryant JM Schürch AC van Deutekom H Harris SR de Beer JL de Jager V. Inferring patient to patient transmission of *Mycobacterium tuberculosis* from whole genome sequencing data. BMC Infect Dis. (2013) 13:110. doi: 10.1186/1471-2334-13-110, PMID: 23446317 PMC3599118

[80] Hakamata M Takihara H Iwamoto T Tamaru A Hashimoto A Tanaka T. Higher genome mutation rates of Beijing lineage of *Mycobacterium tuberculosis* during human infection. Sci Rep. (2020) 10:17997. doi: 10.1038/s41598-020-75028-2, PMID: 33093577 PMC7582865

[81] Comin J Chaure A Cebollada A Ibarz D Viñuelas J Vitoria MA. Investigation of a rapidly spreading tuberculosis outbreak using whole-genome sequencing. Infect Genet Evol. (2020) 81:104184. doi: 10.1016/j.meegid.2020.104184, PMID: 31931260

[82] Lillebaek T Norman A Rasmussen EM Marvig RL Folkvardsen DB Andersen ÅB. Substantial molecular evolution and mutation rates in prolonged latent *Mycobacterium tuberculosis* infection in humans. Int J Med Microbiol. (2016) 306:580–5. doi: 10.1016/j.ijmm.2016.05.017, PMID: 27296510

[83] Ford CB Lin PL Chase MR Shah RR Iartchouk O Galagan J. Use of whole genome sequencing to estimate the mutation rate of *Mycobacterium tuberculosis* during latent infection. Nat Genet. (2011) 43:482–6. doi: 10.1038/ng.811, PMID: 21516081 PMC3101871

[84] Colangeli R Arcus VL Cursons RT Ruthe A Karalus N Coley K. Whole genome sequencing of *Mycobacterium tuberculosis* reveals slow growth and low mutation rates during latent infections in humans. PLoS One. (2014) 9:e91024. doi: 10.1371/journal.pone.0091024, PMID: 24618815 PMC3949705

[85] Yang Z Rosenthal M Rosenberg NA Talarico S Zhang L Marrs C. How dormant is *Mycobacterium tuberculosis* during latency? A study integrating genomics and molecular epidemiology. Infect Genet Evol. (2011) 11:1164–7. doi: 10.1016/j.meegid.2011.02.002, PMID: 21315848 PMC3104100

